# Adverse drug reactions triggered by the common HLA-B*57:01 variant: a molecular docking study

**DOI:** 10.1186/s13321-017-0202-6

**Published:** 2017-03-04

**Authors:** George Van Den Driessche, Denis Fourches

**Affiliations:** Department of Chemistry, Bioinformatics Research Center, North Carolina State University, Raleigh, NC USA

**Keywords:** ADR, HLA variant, Molecular docking, Virtual screening

## Abstract

**Background:**

Human leukocyte antigen (HLA) surface proteins are directly involved in idiosyncratic adverse drug reactions. Herein, we present a structure-based analysis of the common HLA-B*57:01 variant known to be responsible for several HLA-linked adverse effects such as the abacavir hypersensitivity syndrome.

**Methods:**

First, we analyzed three X-ray crystal structures involving the HLA-B*57:01 protein variant, the anti-HIV drug abacavir, and different co-binding peptides present in the antigen-binding cleft. We superimposed the three complexes and showed that abacavir had no significant conformational variation whatever the co-binding peptide. Second, we self-docked abacavir in the HLA-B*57:01 antigen binding cleft with and without peptide using Glide. Third, we docked a small test set of 13 drugs with known ADRs and suspected HLA associations.

**Results:**

In the presence of an endogenous co-binding peptide, we found a significant stabilization (~2 kcal/mol) of the docking scores and identified several modified abacavir–peptide interactions indicating that the peptide does play a role in stabilizing the HLA–abacavir complex. Next, our model was used to dock a test set of 13 drugs at HLA-B*57:01 and measured their predicted binding affinities. Drug-specific interactions were observed at the antigen-binding cleft and we were able to discriminate the compounds with known HLA-B*57:01 liability from inactives.

**Conclusions:**

Overall, our study highlights the relevance of molecular docking for evaluating and analyzing complex HLA–drug interactions. This is particularly important for virtual drug screening over thousands of HLA variants as other experimental techniques (e.g., in vitro HTS) and computational approaches (e.g., molecular dynamics) are more time consuming and expensive to conduct. As the attention for drugs’ HLA liability is on the rise, we believe this work participates in encouraging the use of molecular modeling for reliably studying and predicting HLA–drug interactions.

**Graphical abstract:**
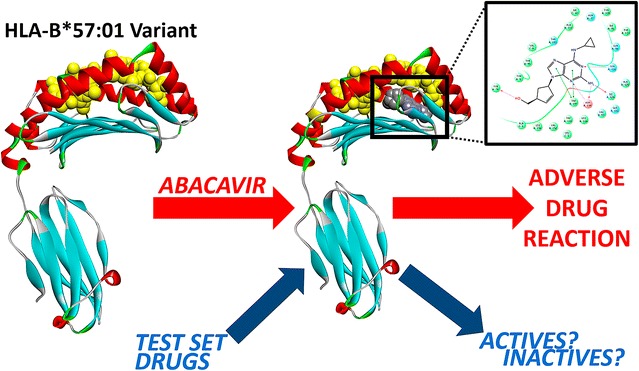
.

**Electronic supplementary material:**

The online version of this article (doi:10.1186/s13321-017-0202-6) contains supplementary material, which is available to authorized users.

## Background

Certain patients develop harmful adverse drug reactions (ADRs) after taking a medication [1]. Unfortunately, these undesired reactions to a drug or its metabolite(s) can potentially be serious and even life threatening. According to the FDA Adverse Event Reporting System (FAERS), over one million cases of ADRs were observed in 2014 and this number is steadily increasing as reporting systems become more accessible to physicians [2]. There are two main classifications of ADRs: *Predictable ADRs* occur due to the pharmacological activity of a drug or its metabolites, whereas *idiosyncratic ADRs* are primarily observed as an immune system response [3, 4]. The major biological pathway capable of triggering such idiosyncratic ADRs is activated by a drug’s direct binding with human leukocyte antigen (HLA) protein variants [4–6].

**Fig. 1:**
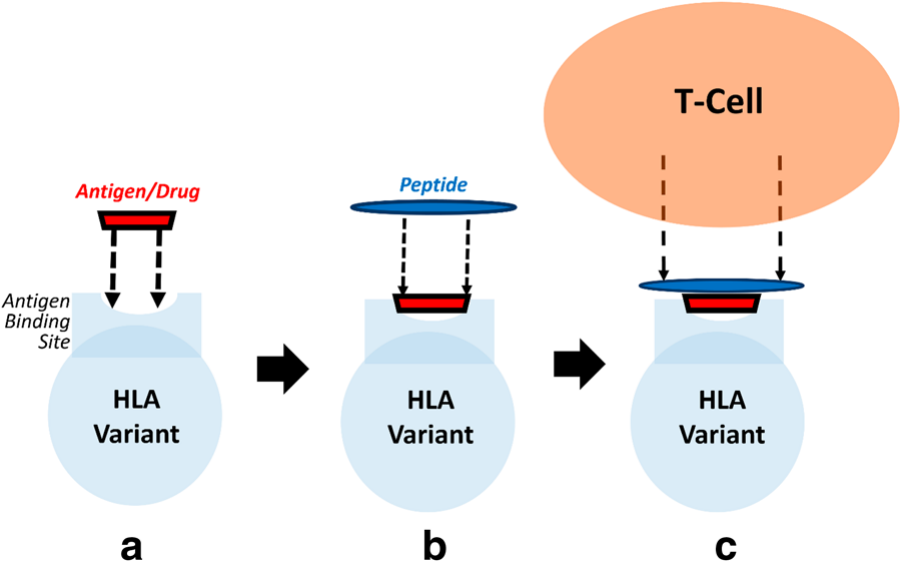
Scheme representation of altered-repertoire binding and signaling mechanism of antigen/drug, HLA-receptor, and peptide for T-cell activation

Class I HLA variants form protein complexes constituted by two main polypeptides and a short self-peptide [7]. As the self-peptide is usually 8–10 residues long, the two polypeptides (approximately 270 residues) form the binding cleft and are also bound to β_2_-microglobulin (β_2_-m), approximately 90–100 residues [7]. Furthermore, the binding cleft can be subdivided into three α-subdomains that consists of two alpha helical regions (α_1_ and α_2_) and a third region (α_3_) that helps in stabilizing the HLA-protein to the cell surface (in addition to the stabilization from β_2_-m) [7]. A groove forms between the α_1_ and α_2_ subdomains that consists of alpha-helical walls and a β-pleated floor where “self” peptides can bind [7, 8]. In case of infection, pathogens (e.g., viral peptides) can bind to HLA instead of the self-peptides and ignite an immune reaction. Indeed, HLA serves as signaling proteins for T cell activation through a variety of proposed mechanisms (e.g., hapten concept, super antigen interactions, pharmacological interactions, altered repertoire) [4, 9]. Overall, the general signaling mechanism involves an antigen (or a drug in the case of ADR) and/or a peptide (endogenous or exogenous) directly binding to the antigen cleft of the HLA, resulting in a signal presentation to T-cell receptors triggering a response of the immune system (Fig. 1). As of today, over 15,000 different HLA-variants have been identified in humans and reported in the IMGT/HLA database [10]. Focusing on ADRs, each variant has hypothetically the potential to form a HLA–drug complex with selective binding interactions. Therefore, the number of possible HLA–drug combinations is enormous and explains why HLA-mediated ADRs are extremely hard to predict and, obviously, rarely observed during clinical trials due to the small number of participants. Furthermore, prioritizing HLA-variants to target for drug screening can be extremely challenging due to the varying frequency of HLA-variant by ethnicity [11].

Even though the vast majority of HLA variants are rare, there are several well-known cases of HLA-mediated ADRs. For instance, carbamazepine (brand name *Tegretol*) is used for the treatment of seizures, but patients carrying the HLA-B*15:02 variant [12, 13] have an increased likelihood to suffer from the Steven-Johnson Syndrome (SJS) or toxic epidermal necrolysis (TEN) [14]. Another well-established example is flucloxacillin (*Floxapen*), a beta-lactam anti-microbial, that can cause typical drug-induced liver injury (DILI) in patients possessing the HLA-B*57:01 variant [15]. Additionally, HLA-B*57:01 is responsible for abacavir hypersensitivity syndrome (AHS) which is a severe, life-threatening ADR occurring in patients prescribed abacavir for treating HIV infection [16].

Clearly, the ability of a drug to bind with a given HLA-variant plays a significant role in determining whether that compound or its metabolite(s) may potentially trigger ADRs in a given subpopulation carrying that particular HLA variant. Therefore, computational approaches able to forecast such HLA–drug molecular interactions reliably could have serious implications in preventing ADRs and thus potentially contribute to the development of precision medicine. Using in silico techniques to predict drugs’ liabilities has now become a common, reliable enough, and cheap enough screening approach, especially for toxicity and ADRs evaluations [17–30]. Typically, forecasting potential ADR involves analyzing the chemical space with chemical similarity techniques [17–20]. Liu et al. recently used a 2D structural alert—based screening for chemical similarity to forecast ADR [17], while Vilar et al. employed a 3D pharmacophore-based similarity search in order to predict ADR [18]. Alternatively, quantitative-structure activity relationship (QSAR) models have been developed in order to forecast drug-induced SJS [19] or DILI [20]. New methods can use a systems chemical biology approach in order to predict drug hepatoxicity through the integration of chemical and biological data [21, 22].

However, there are very few molecular modeling studies in the literature attempting to analyze and predict the molecular interactions between drugs and HLA variants. For instance, Luo et al. [23] modeled the interactions between various HLA variants and some endogenous peptides using a network analysis approach, but the authors did not examine potential drug binding events. Recently, Paul et al. developed two approaches for predicting HLA-Class I and -Class II epitopes using TepiTool [31] and the Immune Epitope Database and Analysis Resonance [32]. Another group developed a very useful, online database compiling all of the known HLA–drug interactions resulting in ADRs (HLADR, http://pgx.fudan.edu.cn/hladr/); however, this database is based solely on measured odds ratios (ORs) obtained from existing literature [24]. Recently, Yang et al. [25] conducted a preliminary molecular docking study on abacavir using AutoDock Vina, but little details were discussed regarding the actual binding mode of the drug. In a *proof*-*of*-*concept* study, we used molecular docking to predict the binding modes of clozapine with several HLA-variants and explore some possible clozapine–HLA interactions [26]. Clozapine (*Clozaril*) is an efficient antipsychotic that may result in agranulocytosis/granulocytosis when a patient has HLA-DQB1 or specific HLA-B variants [26, 27].

Alfirevic et al. [28] attempted to establish a HLA-typed DNA archive that could be used to map DILI events between class I and II HLAs using distance trees. Recently, Schotland et al. [29] attempted to data-mine FAERS reports for SJS/TEN associations with HLA-variants using the Molecular Analysis of Side Effects (MASE) approach. Additionally, Schotland et al. performed a homology docking model of carbamazepine (and several other drugs) at the HLA-B*15:02 variant [29]. This homology model was developed from work previously performed by Wei et al. [30]. Both research groups were able to successfully verify the importance of the ARG62 residue for carbamazepine binding at B*15:02 [29, 30]. Furthermore, using this same homology model Zhou et al. conducted a molecular dynamic simulation exploring the T-cell signaling mechanism of bonded carbamazepine with HLA-B*15:02 [33]. Overall, the literature on the computational modeling of HLA–drug complexes is limited but definitely emerging.

Recently, Metushi et al. conducted a virtual screening of the ZINC database in order to attempt HLA-B*57:01 liable chemicals [34]. The ZINC database contains over 35 million commercially available compounds [35]. Using concatenated FP2 and FP4 structural fingerprints, Metushi et al. conducted a 2D-similarity search of abacavir on 3.5 million compounds from the ZINC database followed by a 3D-similarity search using pharmacophoric features of abacavir [34]. From this initial screening, 54 compounds were identified and selected for molecular docking using the X-ray crystal 3UPR. Next, Metushi et al. identified seven compounds that were tested for HLA-B*57:01 affinity from which acyclovir was identified as a potential candidate [34]. But, when acyclovir was subjected to a CD8^+^ T-cell response assay [36], it was determined that acyclovir did not induce a CD8^+^ T-cell response [34]. This study by Metushi et al. [34] represents a full in silico to in vitro screening for HLA-B*57:01 liable compounds from ZINC.

Developing molecular docking protocols that effectively identify hits can be a challenging undertaking, especially when it comes to the preparation of complex proteins, such as HLA [37]. Moreover, when molecular docking was conducted in published studies involving HLA proteins, it was not specified if a co-binding peptide was present or absent. As such, we believe that it is of the utmost importance that a thorough analysis of molecular docking targeting the HLA-B*57:01 variant be conducted in order to properly identify the limitations of this molecular modeling technique for forecasting a drug’s likelihood to bind a HLA variant and thus potentially cause a HLA-mediated ADR.

**Fig. 2:**
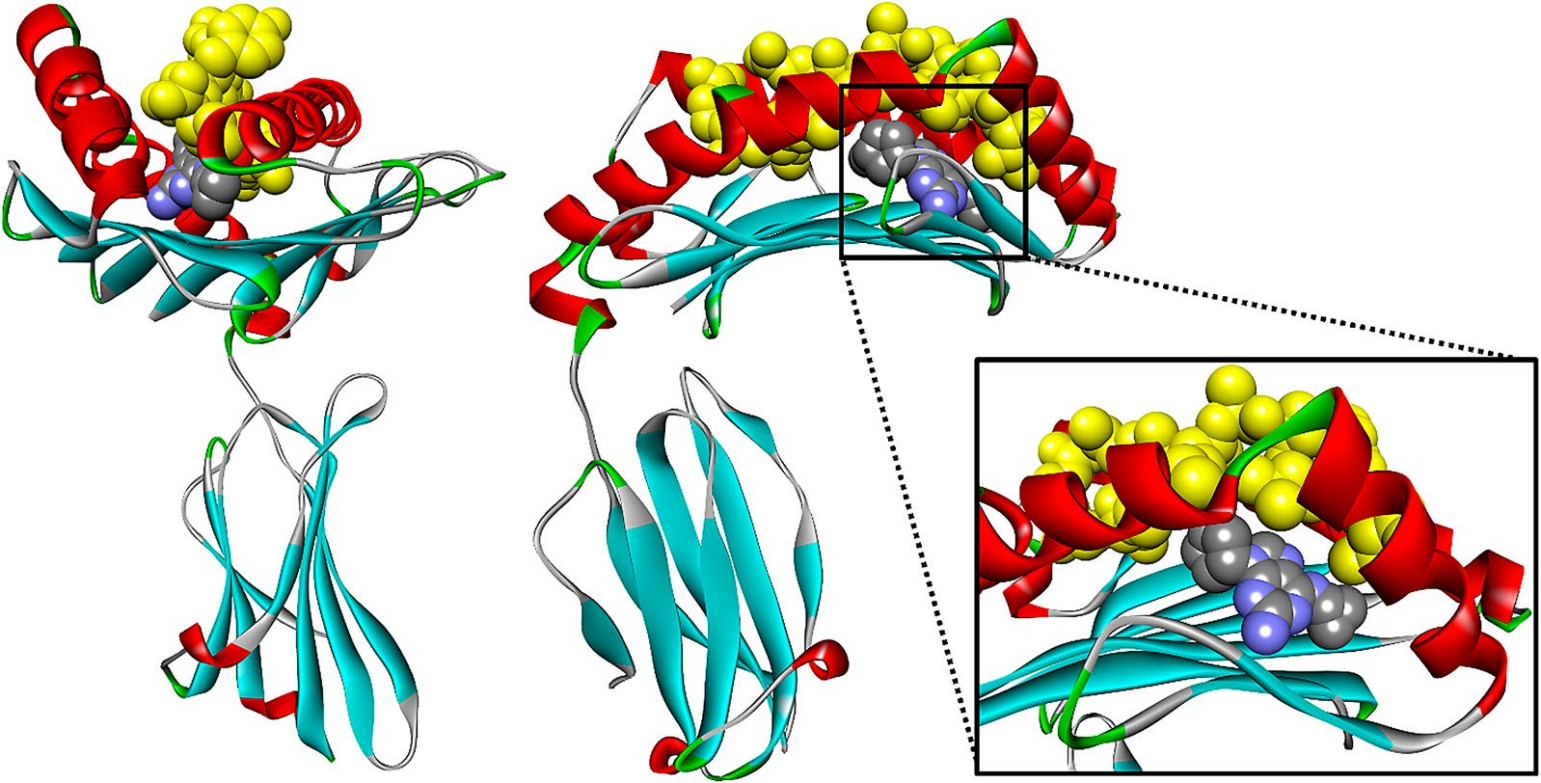
Structure of the HLA-B*57:01 variant in complex with abacavir (represented in *CPK*) and peptide P1 (colored in *yellow*)—PDB code = 3VRI

In this study, we are employing structure-based docking [38] to predict and analyze the molecular interactions between different drugs and the relatively common HLA-B*57:01 variant. First, we decided to focus on abacavir (brand name *Ziagen*) due to the availability of three X-ray crystals (PDB: 3VRI, 3VRJ, and 3UPR) [16, 39]. These X-ray crystals include abacavir and unique co-binding peptides bound in the antigen-binding cleft of HLA-B*57:01 (Fig. 2) [16, 39]. We tested whether molecular docking would be able to obtain native-like peptide–abacavir–HLA complexes. Second, we considered 13 other drugs with known or putative HLA-binding associations resulting in ADR events. We docked them in the antigen-binding cleft of HLA-B*57:01 in presence and absence of an endogenous peptide and critically analyzed their docking scores and binding modes.

Overall, this study underlines the relevance of employing molecular docking for analyzing and predicting HLA–drug interactions. Due to the large number of HLA-alleles [10] and their varying frequency of occurrence by ethnicity [11], the ability to forecast idiosyncratic ADRs is extremely difficult and thus the development of HLA–drug specific virtual screening procedures could become a key component of future precision medicine protocols. Importantly, the goal of this research was to test molecular docking as a computationally inexpensive virtual screening approach for the reliable prediction of critical drug–HLA-B*57:01 interactions triggering ADR events. As such, molecular dynamic simulations were not considered in this study, especially when considering the screening of drugs towards thousands of HLA variants.

## Methods

### Dataset

Three X-ray crystals including abacavir bound to the antigen-binding cleft of HLA-B*57:01 with an endogenous peptide were downloaded from the Protein Data Bank: 3VRI (resolution 1.6 Å, peptide P1, Fig. 2), 3VRJ (resolution 1.9 Å, peptide P2), and 3UPR (resolution 2.0 Å, peptide P3) [16, 39]. The three crystal structures are highly similar and consist of bound abacavir covered by a differing endogenous peptide. In the case of 3VRI and 3VRJ, there are three distinct chains constructing the protein, chain A (275 residues for both crystals), chain B (100 and 99 residues, respectively), and chain C corresponding to the peptides P1 and P2, respectively (10 residues). Crystal 3UPR is a dimer with matching chains A and C (275 residues each), an interlinking chain B and D connecting chains A and C (99 residues), and chains P and Q corresponding to the peptide P3 (9 residues). The binding pocket is located on chain A for 3VRI, 3VRJ, and 3UPR (as well as chain C for 3UPR because it is a dimer).

In order to identify any significant differences between the binding sites of 3VRI, 3VRJ, and 3UPR, we analyzed the overall protein structure, ligand conformation, and co-binding peptides in several different ways. First, a general all-atom alignment was performed between the protein structures as implemented in the Schrodinger Suite [40]. In general, a measured RMSD value can be used to determine structural similarity between closely related proteins (as is the case with our three crystals of the HLA-B*57:01 protein) [41]. Next, a peptide backbone alignment was performed on the three co-binding peptides present in addition to superimposition of bound abacavir from the three crystals. Additionally, overlay similarity scores were calculated between the three structures for the protein, co-binding peptides, and bound abacavir using Discovery Studio [42]. Finally, a binding site-specific alignment was performed using residues within 5 Å of bound abacavir.

Next, the physical chemical characteristics of the binding pocket were explored using SiteMap [43, 44]. SiteMap characterizes the possible binding sites of a protein by analyzing several physical chemical properties, such as size, volume, exposure to solvent, hydrophobic and hydrophilic space, and H-donor/-acceptor ability [43, 44]. Using these descriptors, two scores are generated: Site Score (*Sscore*) and Drugability Score (*Dscore*). A binding pocket that is likely to bind a ligand will have an Sscore greater than 0.8 and a Dscore greater than 0.83 [43, 44]. Using the default SiteMap parameters (6 Å buffer region, a minimum of 15 site points, restrictive hydrophobicity, and a standard grid), the HLA-B*57:01 binding pockets were analyzed under three conditions. First, the binding pocket was analyzed in the presence of the co-binding peptide using abacavir as the reference ligand. Second, the ligand binding environment was analyzed in the absence of co-binding peptide with abacavir as the reference compound. Third, the peptide was used as the reference ligand to map the binding pocket. Analyzing the peptide binding pocket of HLA-B*57:01 under these three conditions afforded a detailed analysis of how the ligand and peptide could impact the binding environment.

Abacavir’s binding mode with HLA-B*57:01 occurs through an altered repertoire mechanism. The altered repertoire binding mechanism occurs when an antigen binds non-covalently to the HLA active site and then, an endogenous or exogenous peptide binds non-covalently across the active site [4, 9, 16, 39]. This prevents the antigen from exiting the binding cleft, while also serving as a signaling trigger to T-cells resulting in an immune system response. As such, the model developed for this study used an altered repertoire binding mechanism. A cartoon schematic of this mechanism is provided in Fig. 1.

The test set of HLA-liable drugs considered in this study is as follows: abacavir (*Zaigen*) [16, 39, 45–47], allopurinol (*Zyloprim*) [48, 49], atorvastatin (*Lipitor*), carbamezapine (*Tegretol*) [12, 13, 29, 30, 50], ciprofloxacin (*Cipro*), clozapine (*Clozril*) [26, 27, 51, 52], fenofibrate (*Triocor*), flucloxacillin (*Floxapen*) [15], methyldopa (*Aldomet*), minocycline (*Minocin*), pazopanib (*Votrient*) [53], sertraline (*Zoloft*), simvastatin (*Zocor*), and ticlopidine (*Ticlid*) [54]. It is worth noting that the drugs abacavir, flucloxacillin, and pazopanib are all HLA-B*57:01 actives, while the rest of the compounds are believed to be inactive towards this particular HLA variant. The set is provided in Table 1 with their respective indications, ADR event, and HLA-association. The set was structurally preprocessed using LigPrep from the Schrodinger Suite [37]. Prior to docking, the therapeutic classes of the test set of ligands were explored in addition to measuring compound similarities. The well-known MACCS key structural fingerprints were employed to compute the pairwise 2D-similarity for the entire test set of compounds [55]. Similarity scores were determined by measuring the Tanimoto coefficients which can be determined using the following equation,

$${\text{T}}_{\text{C}} = \frac{{{\text{b}}_{\text{c}} }}{{{\text{b}}_{1} + {\text{b}}_{2} * {\text{b}}_{\text{c}} }}.$$

Where T_C_ is the tanimoto similarity score, b_C_ are the common bits for both compounds, b_1_ are the bits from molecule one, and b_2_ are the bits from molecule two [56].

**Table 1:** Drugs used to construct test set for docking with their proposed HLA-association

Generic name	Brand name	DBID	Indication	ADR	HLA
Abacavir	*Zaigen*	DB01048	HIV antiviral	Hypersensitivity	B*57:01
Allopurinol	*Zyloprim*	DB00437	Uric acid inhibitor	SCAR	B*58:01
Atorvastatin	*Lipitor*	DB01076	High cholesterol	Hypercholesterolemia cardiac heart disease	DRB1*10:10^a^
Carbamazepine	*Tegretol*	DB00564	Seizures bipolar disorder	SJS/TEN	B*15:02
Ciprofloxacin	*Cipro*	DB00537	Antibiotic	Gastrointestinal irritation	B*50:02^a^
Clozapine	*Clozaril*	DB00363	Antipsychotic	Agranulocytosis	DRB5*02:01
Fenofibrate	*Tricor*	DB01039	High cholesterol	Acute Generalized Exanthematous Pustutosis (AGEP)	A*33:01^a^
Flucloxacillin	*Floxapen*	DB00301	Antibiotic	DILI	B*57:01
Methyldopa	*Aldomet*	DB00968	Anti-hypertensive	N/A	A*33:01^a^
Minocycline	*Minocin*	DB01017	Antimicrobial	Thyroid hyperplasia	B*35:02^a^
Pazopanib	*Votrient*	DB06589	Chemotherapy	DILI ALT concentration increase	B*57:01
Sertraline	*Zoloft*	DB01104	PTSD/OCD	Serotonin syndrome	A*33:01^a^
Simvastatin	*Zocor*	DB00641	High cholesterol	Myalgia arthralgia	B*13:02^a^
Ticlopidine	*Ticlid*	DB00208	Thrombotic stroke	Agranulocytosis aplastic anemia neutropemia	A*33:01

^a^Putative; data not published

### Molecular docking

Prior to the modeling, we conducted a 3D alignment of 3VRI, 3VRJ, and 3UPR in order to evaluate any significant deviations between the protein structures, bound ligand, and peptides. Molecular docking was conducted using the three aforementioned X-ray crystals preprocessed and curated (e.g., removal of water, addition of explicit hydrogens) using the Protein Preparation Wizard from the Schrodinger Suite [37, 57–60]. Missing side chains were generated using Prime [59, 60] while the protonation states of each side chain were generated using EPIK at pH = 7 [57, 58]. Protein minimization was performed using the OPLS3 force field [61–64]. Internal and external receptor grid boxes of 10 × 10 × 10 and 20 × 20 × 20 Å were defined using abacavir bound in the antigen cleft. The optimized structures of the test set were then docked with Schrodinger’s GLIDE software using both SP and XP scoring functions with a rigid protein, flexible ligand, and rigid peptide (when docked with peptide) [65–68]. Due to the presence of a unique peptide for each X-ray crystal, we conducted the docking with and without a peptide covering the solvent-accessible surface of the antigen-binding pocket. Each docking result was analyzed by comparing the docking and eModel scores in addition to the analysis of the drug’s binding mode in the B*57:01 site. The docking score (DS) consists of a sum of the Glide Score, measured from the SP or XP scoring functions, and the measured EPIK state penalty; the eModel score (eM) is a measure of the ‘favorability’ of a docked pose [57, 58, 65–67]. The DS may be used for comparing different ligands, but the eM score is suitable only to rank different conformations of the *same* ligand and should not be used to compare *different* ligands. A drug was considered to be B*57:01 liable (*active*) if the two following empirical thresholds were met: First, the DS had to be at less than or equal to −7 kcal/mol and second, the eM score had to be less than or equal to −50 kcal/mol. These thresholds were previously used in virtual screening protocols for discerning micromolar binders [69–71]. However, these scoring thresholds are specific for our model using GLIDE docking with SP and XP scoring functions and are obviously subject to change depending upon the studied protein, the software and method employed for the virtual screening. Our docking results were also evaluated using accuracy, sensitivity, specificity, positive performance value (PPV), and negative performance value (NPV) [72]; these values can be found in Additional file 1: Table S1.

## Results and discussion

### Alignment of 3VRI, 3VRJ, and 3UPR

**Fig. 3:**
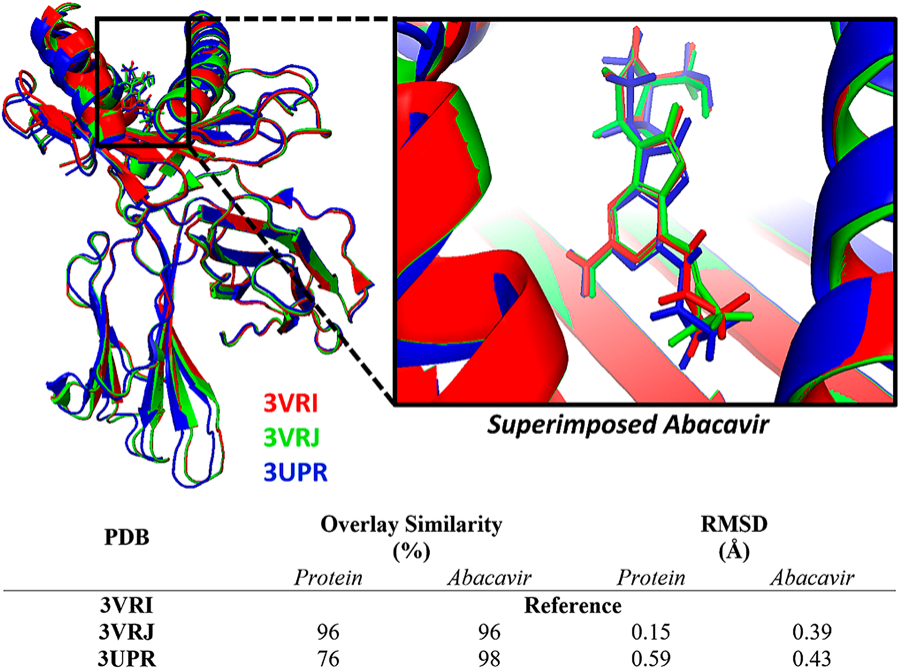
Superimposed structures of HLA-B*57:01 protein and bound abacavir from crystals 3VRI (colored in *red*), 3VRJ (*green*), and 3UPR (*blue*) with measured overlay similarities and RMSD using 3VRI protein and abacavir as the reference structure

We began our analysis by superimposing 3VRI, 3VRJ, and 3UPR and we determined that the most significant differences between these three HLA-B*57:01 crystal structures were related to the co-binding peptides. Indeed, when performing an all-AA residue alignment, 3VRI has a 96% overlay similarity with 3VRJ and an extremely low pairwise RMSD equal to 0.15 Å (see Fig. 3). Meanwhile, the all-AA residue overlay similarity between 3VRI and 3UPR was measured to be 76% with an RMSD of 0.59 Å. The major structural differences between 3VRI and 3VRJ from 3UPR are not related to the actual binding domain (chain A in 3VRI and 3VRJ and chains A and C in 3UPR) but rather the location of the interlinking chains between each crystal (chain B in 3VRI or 3VRJ and chains B and D in 3UPR). Additionally, we aligned the bound conformations of abacavir for all three crystals using 3VRI as the reference structure. We found that the overlay similarities were greater than 95% and measured RMSDs as low as 0.39 and 0.43 Å when compared to 3VRJ and 3UPR respectively. The slight 3D dissimilarity between abacavir’s poses arises due to ring strain of the cyclopropyl group and rotational variation of the hydroxyl group (as illustrated in Fig. 3). When a binding site alignment was performed (using all residues within 5 Å of abacavir), the measured RMSD was less than 0.4 Å for all three crystal structures, which is excellent.

**Fig. 4:**
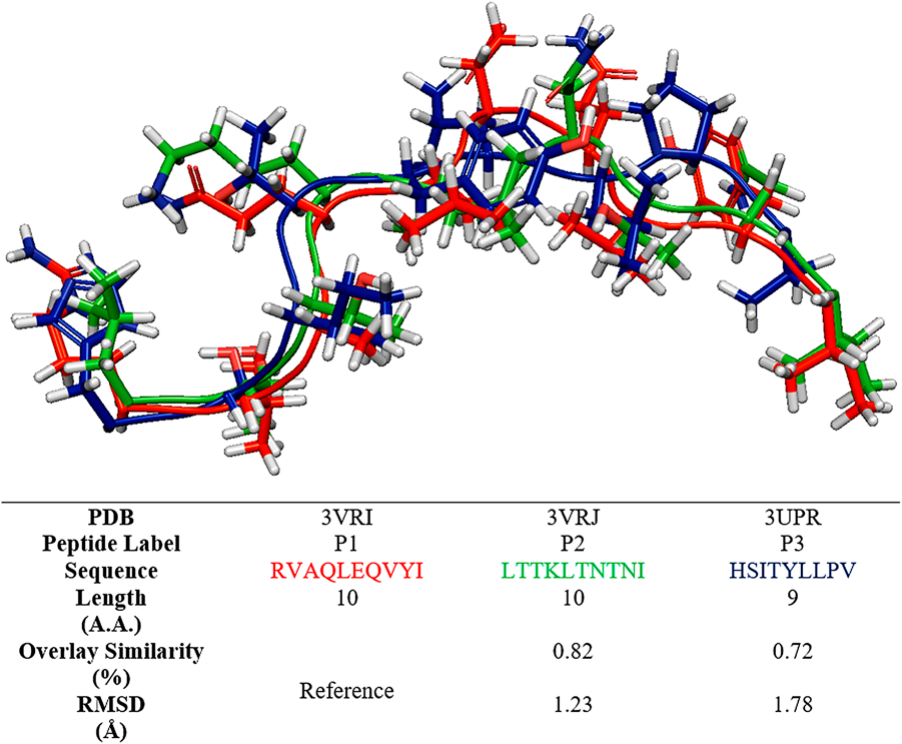
Aligned sequences of peptides P1 (3VRI, *red*), P2 (3VRJ, *green*), and P3 (3UPR, *blue*)

The crystals 3VRI, 3VRJ, and 3UPR each contained a unique co-binding peptide with sequences of RVAQLEQVYI (P1), LTTKLTNTNI (P2), and HSITYLLPV (P3), respectively. After performing a backbone alignment of the peptides, it was determined that the three peptides have a similar binding conformation. When using P1 (from 3VRI) as a reference, the backbone alignment has been measured with an RMSD of 1.23 and 1.78 Å with P2 (3VRJ) and P3 (3UPR) respectively, as shown in Fig. 4. The overlay similarities for all three peptides were greater than 70%. Furthermore, it can be noted that the three peptides have a similar length with both P1 and P2 including 10 amino acid residues and P3 involving 9 residues. This high level of conformational similarity between the three co-binding peptides was not obvious because the amino acid sequence was relatively dissimilar (Fig. 4).

Even though these three peptides contain different residues, they do possess similar physical chemical attributes. For example, with the exception of P2, the ends of each peptide possess a basic residue and a hydrophobic residue (arginine and isoleucine for P1, histidine and valine for P3). Peptide P2 has hydrophobic residues at either extremities (leucine and isoleucine). Additionally, it should be noted that the center of each peptide contains a hydrophobic residue next to a hydrophilic residue. Indeed, peptide P1 has a leucine next to a glutamate at residue positions five and six, P2 has a leucine next to a tyrosine for residues five and six, and P3 has a tyrosine at position six with both a hydrophilic threonine at position four and hydrophobic leucine at position six.

For all three peptides, the AA residues at either end form non-covalent interactions with the binding pocket to anchor the peptide into the pocket. The carbonyl backbone for VAL, TYR, and ILE of P1 serve as H-bond acceptors for the in pocket residues TRP147 and TYR84 in addition to the formation of a salt bridge between ILE carboxylate group and LYS146. On the other end of P1, ARG serves as a H-bond donor with the pocket residues GLU63 and TYR171 while also forming a salt bridge with TYR59. Peptides P2 and P3 are anchored in a similar fashion in crystals 3VRJ and 3UPR. Importantly, the centroid of each peptide does not form interactions with binding pocket due to displacement by abacavir. The 2D-binding modes of peptides P1, P2, and P3 are provided in Additional file 1: Fig. S1.

Furthermore, the physical binding environment of the binding pocket was evaluated using SiteMap [43, 44]. The binding pocket for each crystal was evaluated under three conditions: (1) abacavir was used as the reference ligand with peptide present, (2) abacavir was used as the reference ligand in the absence of a co-binding peptide, and (3) the co-binding peptide was used as the reference ligand with abacavir present. Under conditions 1 and 2, the measured Sscore and Dscore were ranging between 1.1 and 1.3 indicating that the binding environment is extremely favorable (>0.8). Overall, condition 2 afforded slightly lower Sscore and Dscores, which is most likely due to increased solvent exposure due to the missing co-binding peptide. Interestingly, when the co-binding peptide was used as the reference ligand, the Sscore and Dscore were significantly lowered to a range of 0.8–1.1. Additionally, SiteMap identified two binding locations under the third condition (at either end of the peptide), while the center of the peptide was excluded from the binding surface. The observation of two binding pockets occurs as a result of the altered repertoire binding mechanism of abacavir with HLA-B*57:01: Abacavir displaced the center of the peptide from binding with the pocket. All SiteMap-generated binding surfaces are provided in Additional file 1: Figure S2.

### Self-docking of abacavir in 3VRI with (+) and without (−) the presence of a co-binding peptide

The next step of our analysis was dedicated to the self-docking of abacavir in both the presence and absence of a co-binding peptide in B*57:01. To do so, we removed the native pose of abacavir from 3VRI, then we re-docked abacavir using both Glide SP and XP scoring functions. This self-docking procedure was conducted in order to test whether molecular docking could accurately reproduce the native binding mode of abacavir and investigate the significance of the co-binding peptide. To do so, we aligned the highest scoring conformation of self-docked abacavir with the native pose of abacavir from 3VRI. Self-docking without P1 was also performed; however, there is limited existing data about the potential binding mode of abacavir without peptide [16, 39]. When attempting to solve the X-ray crystals 3VRI and 3VRJ, Illing et al. [16] used molecular docking to probe the binding cleft of HLA-B*57:01 to assist their crystallization procedure. Similarly, when Ostrov et al. [39] solved for the 3UPR crystal, molecular docking was employed to select an optimized co-binding peptide for crystallization. Next, both the measured DS and eM scores of self-docked abacavir (with and without P1) were analyzed followed by the description of the molecular interactions between abacavir and HLA-B*57:01.

**Fig. 5:**
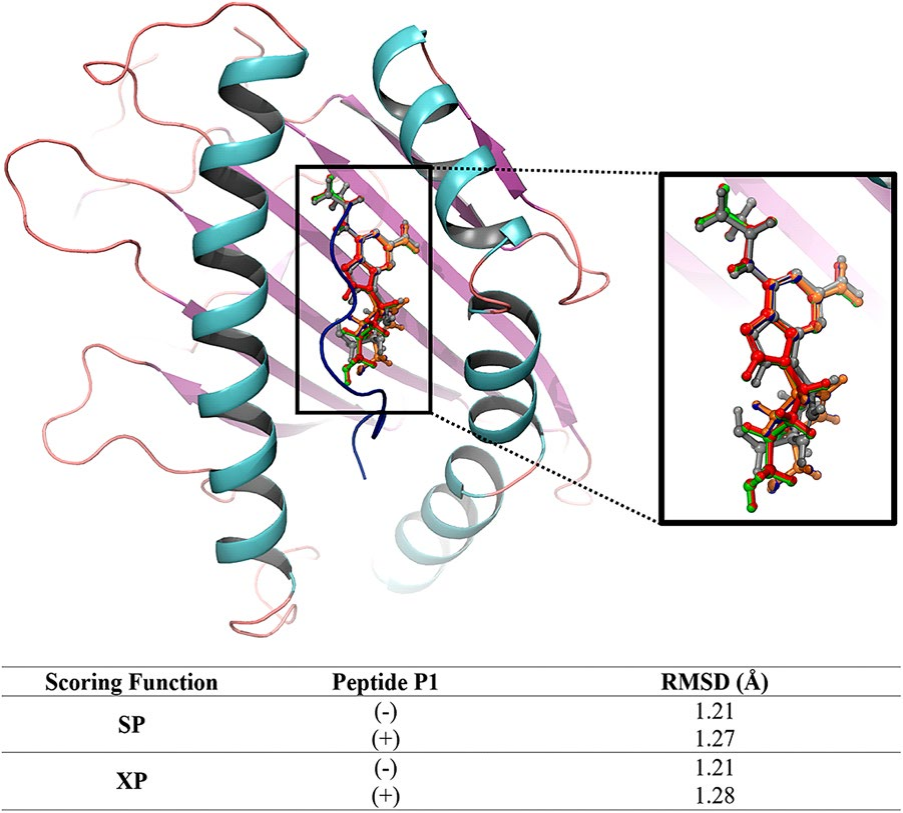
Self-docked abacavir with measured RMSD using crystal 3VRI. 3VRI native abacavir is shown in *gray*, abacavir using SP without P1 is shown in *blue*, abacavir using SP with P1 is shown in *red*, abacavir using XP without P1 is shown in *orange*, and abacavir using XP with P1 is shown in *green*

Surprisingly, the self-docked abacavir had a measured RMSD of about 1.2 Å for 3VRI with and without P1, regardless if the SP or XP scoring function was used. As illustrated in Fig. 5, this variation in RMSD was determined to be from the rotation of the hydroxyl group and ring strain from abacavir’s cyclopropyl group. Additional self-docking alignments were conducted for both 3VRJ and 3UPR. The most notable difference for these compounds was the observance that docked abacavir in the absence of P2 or P3 would rotate 180° placing the hydroxyl group and cyclopropyl group at opposite ends of the binding pocket from the native crystal. However, when P2 or P3 were present, the predicted binding mode matched the native crystal. These results are provided in Additional file 1: Figures S3 and S4 for 3VRJ and 3UPR, respectively. This difference in binding orientation in the absence of co-binding peptide could occur from three possibilities: First, the X-ray crystals contain peptides so the actual binding mode of abacavir without peptide is unknown. Second, the binding pocket has similar residues allowing different orientations of abacavir to bind in the absence of peptide. Third, there may be two equally stable orientations of abacavir present in the binding pocket in the absence of a co-binding peptide.

 The measured DS and eM scores for self-docked abacavir are summarized in Table 2. For this study, a compound is considered as a B*57:01 binder if it affords a DS less than −7 kcal/mol and an eM score less than −50 kcal/mol [69–71]. Importantly, self-docked abacavir was found to be active for all three protein structures with and without peptides P1, P2, and P3. Furthermore, the DS were found to be within a 1 kcal/mol variation for both SP and XP scoring functions and across crystal structures. Moreover, the presence of a co-binding peptide was found to stabilize the DS by approximately 2 kcal/mol in all cases. We found significantly more variation regarding eM scores; the observed differences between scoring functions were ranging from 0.7 to 4.7 kcal/mol, while the differences between crystals were ranging from 0.2 to 7 kcal/mol. The larger the variance in eM scores, the more diverse the conformational poses of abacavir.

**Table 2:** DS and eM scores reported as absolute values for abacavir—B*57:01 docking with crystals 3VRI, 3VRJ, and 3UPR in the presence and absence of peptides P1, P2, and P3 using the SP and XP scoring functions

	3VRI		3VRJ		3UPR	
	(−) P1	(+) P1	(−) P2	(+) P2	(−) P3	(+) P3
SP						
Docking (kcal/mol)	−8.27	−10.46	−8.46	−9.64	−8.24	−9.51
eModel (kcal/mol)	−62.7	−78.5	−64.3	−79.8	−65.3	−78.3
XP						
Docking (kcal/mol)	−7.99	−10.35	−7.38	−9.06	−7.77	−9.22
eModel (kcal/mol)	−58.0	−74.9	−65.0	−76.5	−62.4	−78.9

**Fig. 6:**
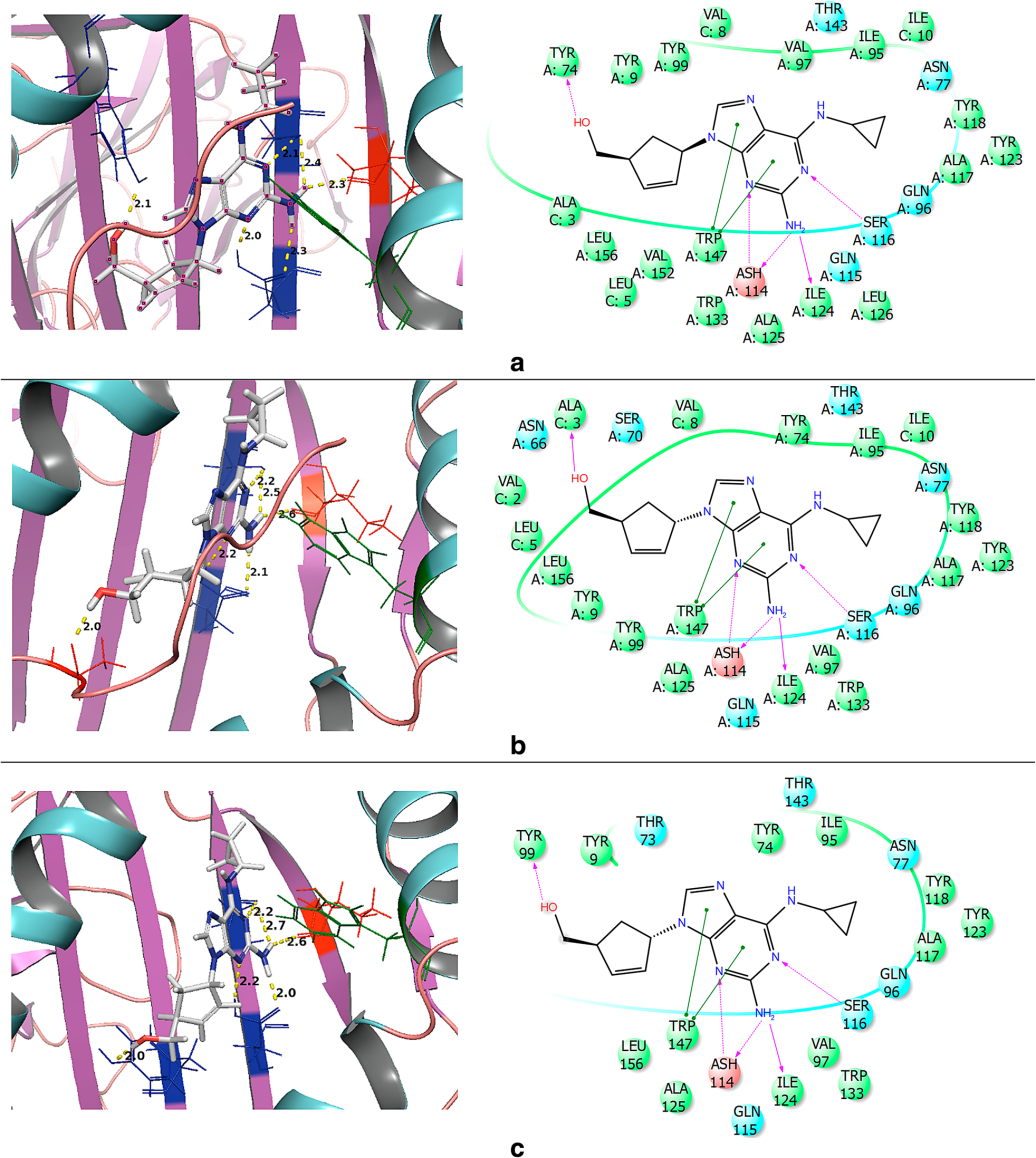
Binding mode interactions of abacavir at B*57:01 using 3VRI: **a** Native abacavir binding mode from X-ray crystal 3VRI with P1; **b** Self-docked abacavir using SP scoring function with P1; **c** Self-docked abacavir using SP scoring function without P1. B*57:01 represented as follows: Helix (*cyan*), sheet (*magenta*), and loops (*salmon*). Molecular interactions colored as follows: side chain H-bonding (*dark blue*), backbone H-bonding (*red*), and π–π stacking (*green*)

Lastly, the binding modes were examined to elucidate the impact the co-binding endogenous peptide could have upon the actual DS and eM scores (DS was stabilized by approximately 2 kcal/mol while the eM score was stabilized by 10–15 kcal/mol in the presence of peptide). The binding mode of abacavir with and without P1 generated using the SP scoring function is represented in Fig. 6. Figure 6a shows the binding mode of native abacavir with P1 (3VRI) and it was observed that the terminal hydroxyl group is rotated into the binding pocket and undergoes H-bonding with the TYR74 residue (R_OH—TYR74_ = 2.1 Å). However, when abacavir is docked without P1, the hydroxyl group still engages in H-bonding with a tyrosine residue, but it is now TYR99 (R_OH—TYR99_ = 2.0 Å) as shown in Fig. 6b. However, when P1 is present, the hydroxyl group is rotated away from the binding pocket and H-bonds with the peptide backbone (carbonyl) of ALA3 (R_OH—ALA3_ = 2.0 Å) as shown in Fig. 6c. Overall, we determined that the H-bonding (ASH114, SER116, and ILE124) and π–π stacking (TRP147) between abacavir and the B*57:01 binding pocket involved the same amino acid interactions in both presence and absence of the peptide except for the terminal hydroxyl group of abacavir. The binding modes for abacavir with 3VRJ and 3UPR are provided in Additional file 1: Figures S5 and S6, respectively. Thus, Glide was able to accurately reproduce the native binding mode of abacavir with HLA-B*57:01 and afforded good DS and eM scores. Performing molecular docking using the crystals 3VRI, 3VRJ, and 3UPR should thus be able to forecast meaningful interactions between HLA-B*57:01 and drugs from the test set. The results of docking using 3VRI and P1 are included in the manuscript, while the results of docking with 3VRJ (P2) and 3UPR (P3) are provided in the Additional file 1.

### Docking a set of ADR-causing drugs

The test set of ADR-causing drugs with known or putative HLA-binding profiles (Table 1) were docked using both SP and XP scoring functions. Upon inspection, we were able to determine that the test set of drugs used in this study had distinct therapeutic classes and low 2D chemical similarities. All of the drugs from the test set had distinct therapeutic classes with the exception of flucloxacillin and minocycline, which were both antibacterial agents. Furthermore, when the MACCS fingerprints [55] were calculated, the Tanimoto similarities [56] were quite distinct with the highest similarity (0.63) between the drugs atorvastatin and ciprofloxacin, and clozapine and ticlopidine, respectively. Abacavir and fenofibrate had the lowest measured structural similarity of 0.15. Surprisingly, the drugs abacavir, flucloxacillin, and pazopanib were all dissimilar with Tanimoto’s ranging from 0.35 to 0.40 even though these drugs are HLA-B*57:01 liable [15, 16, 39, 53]. All pairwise Tanimoto similarities are provided in Additional file 1: Table S2.

Virtual screening was conducted using the three X-ray crystals 3VRI, 3VRJ, and 3UPR in the presence and absence of peptide. Herein, we reported the results of docking using crystal 3VRI with and without peptide P1 (the results pertaining to docking performed with 3VRJ (P2) and 3UPR (P3) are provided in the Additional file 1). Statistical measures of our model’s ability to forecast HLA-B*57:01 liable drugs have been provided in Additional file 1: Table S1. Interestingly, our model performed very well in the absence of peptides P1, P2, and P3 [SEN = 0.67 (SP) and 1.00 (XP)], but when the peptide was included in docking, there was a significant decrease in our model’s ability to predict true positives (SEN = 0.33 for SP and XP). However, our model’s ability to forecast true negatives was extremely good as indicated by the high specificity (0.73–0.82 for SP and XP without peptide and 0.91–1.00 for SP and XP with peptide). The accuracy, positive prediction value (PPV), and negative prediction value (NPV) were also measured and are provided in Additional file 1: Table S1.

**Fig. 7:**
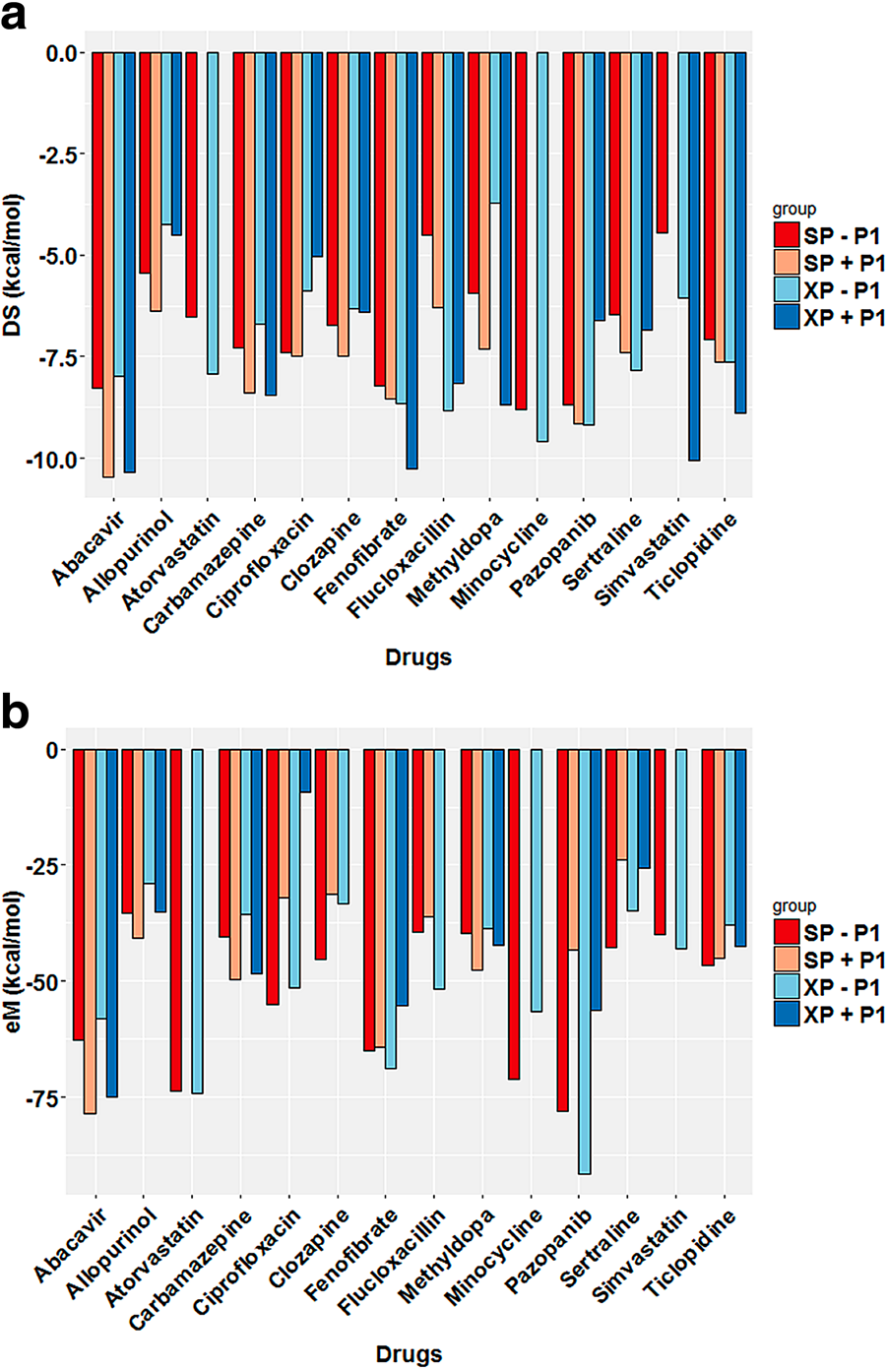
Docking score (**a**) and eModel (**b**) distributions for the test set of drugs. Scores obtained with 3VRI are reported as absolute values with thresholds at 7 kcal/mol (docking score) and 50 kcal/mol (eModel). SP results without P1 are colored in *red*, SP with P1 shown in *maroon*, XP without P1 shown in *light blue*, and XP with P1 shown in *dark blue*

The DS results are plotted in Fig. 7a for the entire test set, while eM scores are plotted in Fig. 7b. It should be noted, that though each score is represented in a separate image for simplicity in viewing, the best criteria for an active compound is that BOTH the DS and eM scoring thresholds be met (DS ≤ −7 kcal/mol and eM ≤ −50 kcal/mol, respectively) [69–71]. Using this dual threshold requirement, it was observed that the drugs abacavir, fenofibrate, and pazopanib all met the criteria to be B*57:01 active drugs. The results pertaining to abacavir have previously been discussed in section "Self-Docking of abacavir in 3VRI with (+) and without (-) the presence of a co-binding peptide". Interestingly, fenofibrate, a drug believed to bind the HLA-A*33:01 variant (Table 1), was forecasted as a HLA-B*57:01 active compound by our model. This result suggests that our model may be inappropriately classifying fenofibrate as an HLA-B*57:01 liable drug; however, there are instances where drugs have shown promiscuity towards multiple variants (consider carbamazepine with HLA-A*31:01 and HLA-B*15:02) [12, 50]. As such, future studies should be performed using molecular dynamics to analyze the binding mode and binding affinity of fenofibrate at both A*33:01 and B*57:01 variants. Reassuringly, Glide was able to accurately forecast the B*57:01 active drug pazopanib [53]. The binding results of pazopanib are discussed in section "Additional focus on known HLA-B*57:01 actives: Flucloxacillin and Pazopanib".

Interestingly, our model identified allopurinol and clozapine as two inactive compounds, i.e., failed to meet either DS or eM score criteria at the B*57:01 variant. Allopurinol is a HLA-B*58:01 active compound [48, 49] and was not expected to be active at the B*57:01 variant. Previous work had suggested that clozapine might be a HLA-B*39 or -B*57:01 active drug, but it was unclear which specific B-variant was preferred [26, 27]. In our current model, clozapine afforded a DS above the threshold of −7 kcal/mol and an eM greater than −50 kcal/mol providing further evidence that clozapine is not a B*57:01 binder. In some cases, our model produced conflicting results that would predict a drug as being inactive or active under varying conditions. For example, as shown in Fig. 7a, carbamazepine afforded DS well below the threshold (with the exception of G_XP_ without P1); however, the measured eM score of carbamazepine were greater than our −50 kcal/mol threshold indicating that carbamazepine was not an HLA-B*57:01 liable drug (Fig. 7b).

In other instances, the model identified a drug as a B*57:01 active in the absence of peptide, but when the P1 peptide was present in the cleft, the drug would fail to meet either DS, eM, or both criteria. The drugs atorvastatin (HLA-DRB1*10:10 active), ciprofloxacin (HLA-B*50:02), and minocycline (HLA-B*35:02) were among these cases (Table 1). It was also observed that for some drugs, such as flucloxacillin, the use of SP or XP scoring function influenced the models prediction. The DS and eM scores for 3VRJ and 3UPR with the test set are provided in Additional file 1: Figures S7 and S8, respectively.

Pearson correlation coefficients were calculated for DS and eM scores obtained by the three X-ray crystal docking systems (Additional file 1: Tables S3, S4). The DS results without peptide showed a significant improvement in fit when the XP scoring function was used between 3VRI, 3VRJ, and 3UPR (0.71 ≤ R_SP(−)P_ ≤ 0.88 and 0.92 ≤ R_XP(−)P_ ≤ 0.98); however, when docking was performed in the presence of peptides P1, P2, or P3, there was more fluctuation in the results (0.57 ≤ R_SP(+)P_ ≤ 0.87 and 0.70 ≤ R_XP(+)P_ ≤ 0.74). Interestingly, when we compared eM scores, the similarity between docking grids appeared to be more dependent upon the scoring function employed as opposed to the influence of the peptide. When the SP function was used, the crystals showed excellent agreement in the absence of peptide, but in the presence of peptide there was more variation between the systems (0.73 ≤ R_SP(−)P_ ≤ 0.84 and 0.60 ≤ R_SP(+)P_ ≤ 0.91). Yet, when the XP function was used the overall fit improved in the absence and presence of peptide (0.60 ≤ R_XP(−)P_ ≤ 0.91 and 0.67 ≤ R_XP(+)P_ ≤ 0.83). For both DS and eM scores, the presence of peptide lowered the correlation, because not all of the test set drugs had a favorable conformation in the binding pocket with peptide. The discrepancy observed between scoring functions for eM correlations could result from differences between the SP and XP scoring functions in calculating conformational energy of the binding drug [65–68]. Importantly, the eM score was not used to compare results between different sets of ligands, but to measure how the SP and XP scoring functions performed when different crystals (3VRI, 3VRJ, and 3UPR) under different stresses (the presence or absence of peptides P1, P2, and P3) were used. Essentially, this provides a baseline for measuring the differences between our systems.

**Fig. 8:**
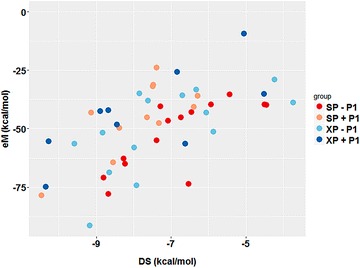
eModel versus docking score plot for test set of drugs. SP without P1 results are shown in *red*, SP with P1 shown as *maroon*, XP without P1 shown in *light blue*, and XP with P1 shown in *dark blue*

Next, we plotted eM scores against DS in order to emphasize the compounds that surpassed both DS and eM score thresholds, as shown in Fig. 8. Interestingly, in the absence of P1, pazopanib is the best scoring drug (DS_XP_ −9.14 kcal/mol, eM_XP_ = −91.55), but when P1 is present abacavir is the best scoring drug. One compound not shown in Fig. 8 is simvastatin, which had an extremely favorable DS of −10.07 kcal/mol when using the XP scoring function with P1. However, the measured eM of simvastatin was highly unfavorable (eM ≥ 0 kcal/mol). We believe this may show the limitation of Glide scoring functions for docking these complex tripartite systems. When molecular docking is performed, the protein is treated as a rigid system while the ligand is a flexible system. In this tripartite system, the co-binding peptide needs to be accounted for in the docking procedure. Here, the peptide is treated as a rigid system similarly to the protein. Clearly, molecular dynamic simulations will be needed in order to explore how the co-binding peptide adjusts to the presence of different drugs. Furthermore, this anomaly may present evidence that our model can only accurately handle ligands that share similar 3D-characteristics with abacavir (size, shape, functional groups, etc.). Future studies will incorporate molecular dynamics to investigate the dynamic relationships between the HLA-binding pocket, drug, and peptide.

There were two top scoring drugs when docking with 3VRJ (see Additional file 1: Figures S9): abacavir docked using the SP function with peptide P2 and pazopanib docked using the XP function without peptide P2. When docking was performed with 3UPR, the top performing drug was again abacavir (the SP and XP scoring functions with peptide P3 both yielded the best DS and eM scores in this case). The plot of eM versus DS for 3UPR is provided in Additional file 1: Figures S10.

We should underline the results obtained in our docking study seem to be dependent on the composition of the co-binding peptides and this can be seen as a clear limitation. Thus, more analysis would be needed to study whether peptide P1 is rather specific to abacavir and therefore does not allow favorable interactions for the binding modes of other drugs (such as flucloxacillin or pazopanib which are both HLA-B*57:01 liable, but were predicted as inactive in the presence of peptide). Thus, HLA-variants may bind drugs with drug-specific (or class-specific) peptides that could significantly enable, boost, and thus impact their binding modes. Another possibility is related to the fact that P1 probably adopts a specific conformation favorable for abacavir binding. It means that other drugs may bind differently in the B*57:01 pocket and these binding modes are not favored in P1’s conformation from 3VRI. Thus molecular dynamic simulations [73] are needed to test and potentially confirm this hypothesis at the case by case level. Of course, MD simulations cannot be used for screening purposes, especially considering a large collection of drugs (e.g., DrugBank) towards thousands of HLA variants. Therefore, molecular docking should still be considered as the main approach for high throughput HLA–drug screening.

### Additional focus on known HLA-B*57:01 actives: flucloxacillin and pazopanib

In addition to abacavir, the test set of compounds contained two other drugs, flucloxacillin and pazopanib, known to bind HLA-B*57:01 and potentially causing drug-induced liver injury (DILI) [15, 53]. Glide was able to successfully forecast pazopanib as a B*57:01 active drug with DS (DS_SP_ = −8.7 kcal/mol, DS_XP_ = −9.2 kcal/mol) and eM scores (eM_SP_ = −78.0 kcal/mol, eM_XP_ = −91.5 kcal/mol) well above the threshold in the absence of P1. Notably, the presence of P1 had a significant impact upon the DS and eM scores for pazopanib. When the SP function was used, pazopanib’s DS surpassed the threshold (DS_SP_ = −9.1 kcal/mol), while the eM score failed to meet it (eM_SP_ = −43.2 kcal/mol). However, when the XP function was used the opposite phenomenon was observed with the DS failing the threshold (DS_XP_ = −6.6 kcal/mol) and the eM score surpassing it (eM_XP_ = −56.3 kcal/mol). As a result, the binding mode of pazopanib was investigated for SP and XP scoring functions with and without peptide P1.

**Fig. 9:**
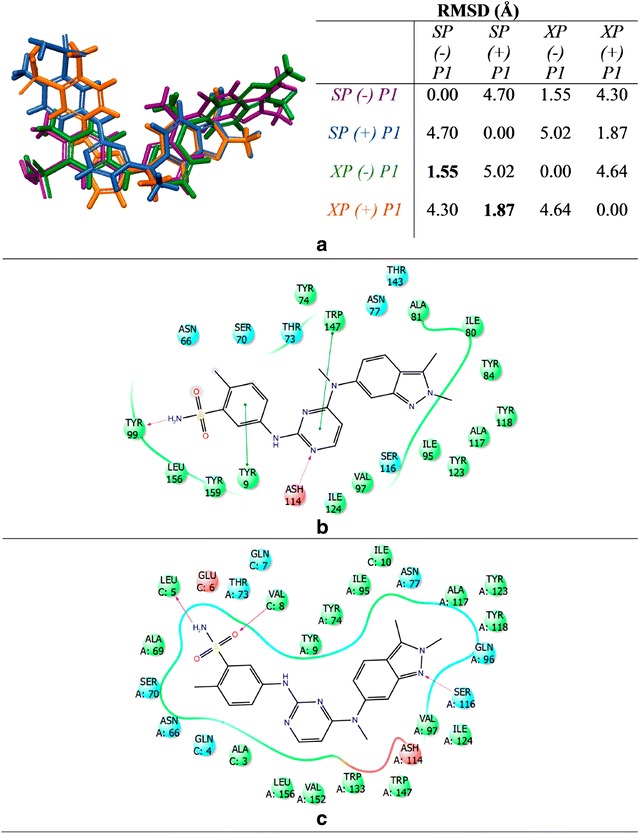
Binding mode of pazopanib in 3VRI. **a** Alignment of pazopanib from SP and XP scoring functions with and without peptide P1. SP (−) P1 is shown as *purple*, SP (+) P1 is shown as *blue*, XP (−) P1 is shown as *green*, and XP (+) P1 is shown as *orange*. **b** 2D binding mode from SP (−) P1. **c** 2D binding mode from SP (+) P1

We aligned the SP and XP best docked poses and found that the binding modes of pazopanib were the same (Fig. 9a). We then found that pazopanib adopts at least two main conformations in the binding site. In the absence of P1, pazopanib favors a linear conformation (Fig. 9b) while in the presence of P1 a distinct structural curvature is formed (Fig. 9c). The linear conformation adopted in the absence of P1 (Fig. 9b) may occur as a result of optimizing ligand-residue interactions in the binding pocket that occur with H-bonds formed between TYR99 and the sulfonamide functional group, and ASH114 and a N-lone pair from the pyrimidine moiety. Additionally, there is some π–π stacking observed between TYR9 and the *p*-methyl-*m*-sulfonamide-benzyl group as well as some stacking between TRP147 and the pyrimidine moiety. In the presence of P1, pazopanib adopts a curved conformation. There are two possible causes for this change in binding conformations (from linear to curved). First, some ligand–peptide interactions were not available in the absence of P1. Furthermore, the π–π stacking observed in the absence of P1 is no longer present as shown in Fig. 9c. The key peptide residues interacting with pazopanib are LEU5 and VAL8, which are H-bonding through the backbone of P1 with the sulfonamide functional group. There is also H-bonding occurring in the pocket between SER116 (not a residue of the peptide) and the N-lone pair from the indazole moiety. Again, molecular dynamic simulations are needed to further investigate how the peptide influences the conformation of pazopanib.

**Fig. 10:**
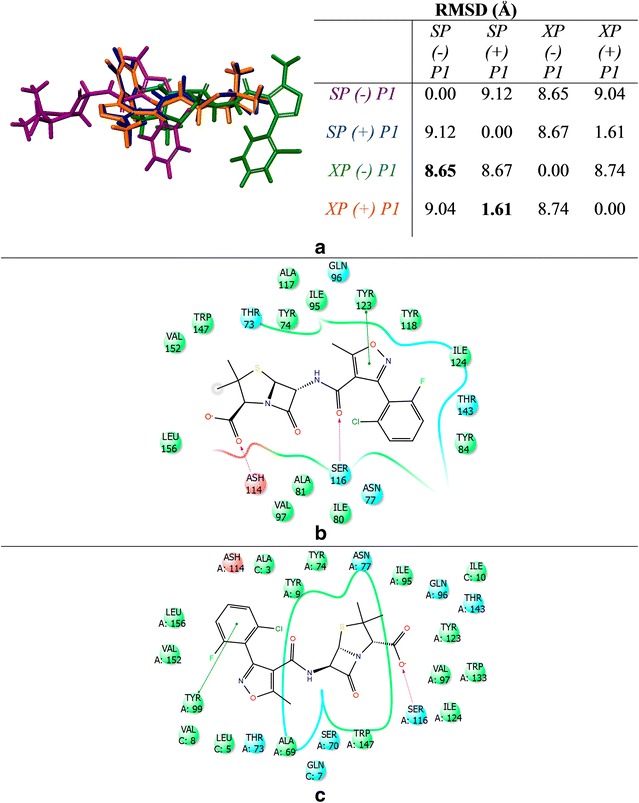
Binding mode of Flucloxacillin in 3VRI. **a** Alignment of flucloxacillin from SP and XP scoring functions with and without peptide P1. SP (−) P1 is shown as *purple*, SP (+) P1 is shown as *blue*, XP (−) P1 is shown as *green*, and XP (+) P1 is shown as* orange*. **b** 2D binding mode found from XP (−) P1. **c** 2D binding mode found from XP (+) P1

The binding mode of flucloxacillin was also studied (Fig. 10). Interestingly, we found that flucloxacillin could adopt four different metastable conformations in the B*57:01 pocket and these conformations are significantly different as indicated by the large RMSD values (Fig. 10a). We observed that in the absence of P1, flucloxacillin showed significant conformational variations with pairwise RMSDs greater than 8 Å regardless of the scoring function. However, when P1 was present for SP and XP, the RMSD was found to be as low as 1.64 Å. This result indicates that the binding mode of flucloxacillin may be highly dependent on the co-binding peptide. Curiously, each conformer was the top-scoring conformation for flucloxacillin from each of the conditions tested (SP with/out peptide and XP with/out peptide). Interestingly, the measured DS and eM scores from the SP scoring function were well above the threshold in the absence of P1 (DS_SP_ = −4.5 kcal/mol, eM_SP_ = −39.6 kcal/mol) and presence of P1 (DS_SP_ = −6.3 kcal/mol, eM_SP_ = −36.1 kcal/mol) indicating that flucloxacillin would be inactive for B*57:01. However, when the XP scoring function was used, the DS and eM scores were significantly lower than the threshold in absence of P1 (DS_XP_ = −8.8 kcal/mol, eM_XP_ = −51.8 kcal/mol), but in the presence of P1 the measured DS passed our threshold while the eM score was greater than zero (G_XP_ = −8.1 kcal/mol). This result indicates that flucloxacillin is a B*57:01 active compound in the absence of P1. One possible reason for this divergence in the prediction results may arise from the fact that the XP scoring function could more accurately account for the flexible β-lactam subunit in flucloxacillin [65–68]. Another possibility is that the ideal binding location of flucloxacillin is located in a different region of the binding pocket (as indicated by superimposition). These peculiar results for flucloxacillin and simvastatin (XP + P1) may indicate a severe limitation of using molecular docking for such complicated three part systems (protein, ligand, and co-binding peptide).

Finally, the binding mode of flucloxacillin was further investigated using the XP scoring function with and without peptide P1 (see Fig. 10b, c). There are very significant differences in the binding mode indicating that the binding location in B*57:01 most likely occurs in a different region of the pocket than where the docking grid was generated using abacavir. More specifically, in the absence of P1, H-bonding is observed between ASH114 and the carboxylate group and between SER116 and the amide linker; π–π stacking also occurs between TYR123 and the isoxazol moiety (Fig. 10b). However, when docking with P1 the ligand-residue interactions change drastically (in addition to the global orientation of the ligand). As shown in Fig. 10c, the H-bonding of SER116 now occurs with the carboxylate group, whereas π–π stacking occurs between TYR99 and the 2-chloro-6-fluoro-phenyl group. Consequently, the position of flucloxacillin has shifted in the protein pocket. These significant variations in flucloxacillin’s binding modes indicate that molecular dynamic studies will be needed in order to further investigate the potential binding mode(s) between B*57:01 and flucloxacillin.

## Conclusions

**Fig. 11:**
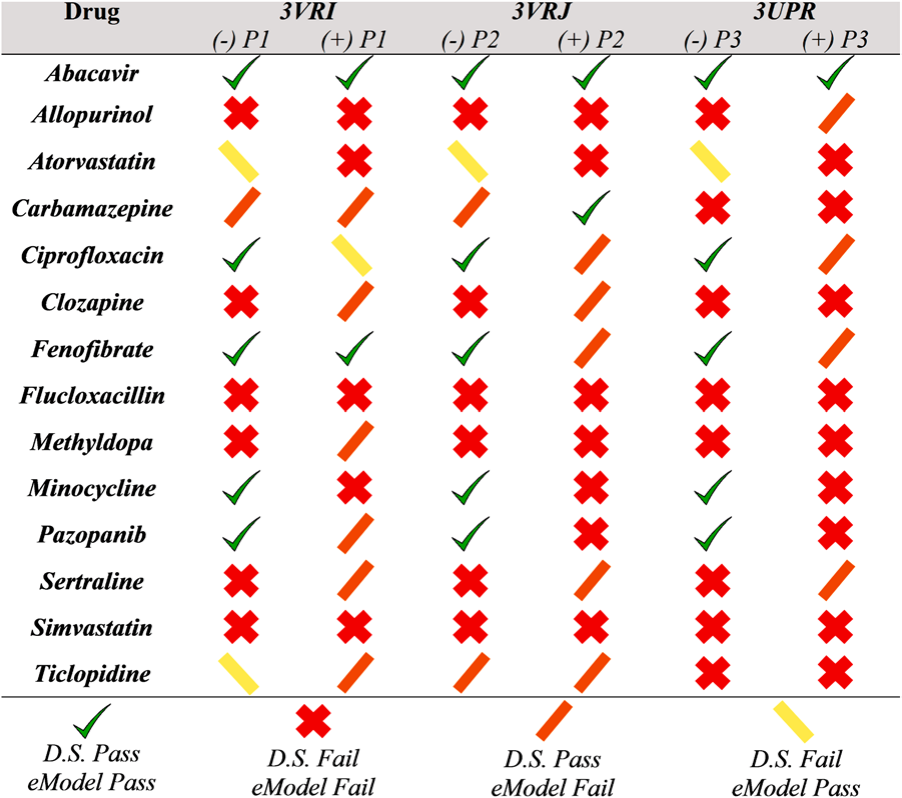
Docking results summary across all three crystals (PDB: 3VRI, 3VRJ, 3UPR) using the SP scoring function with and without peptides P1, P2, and P3

**Fig. 12:**
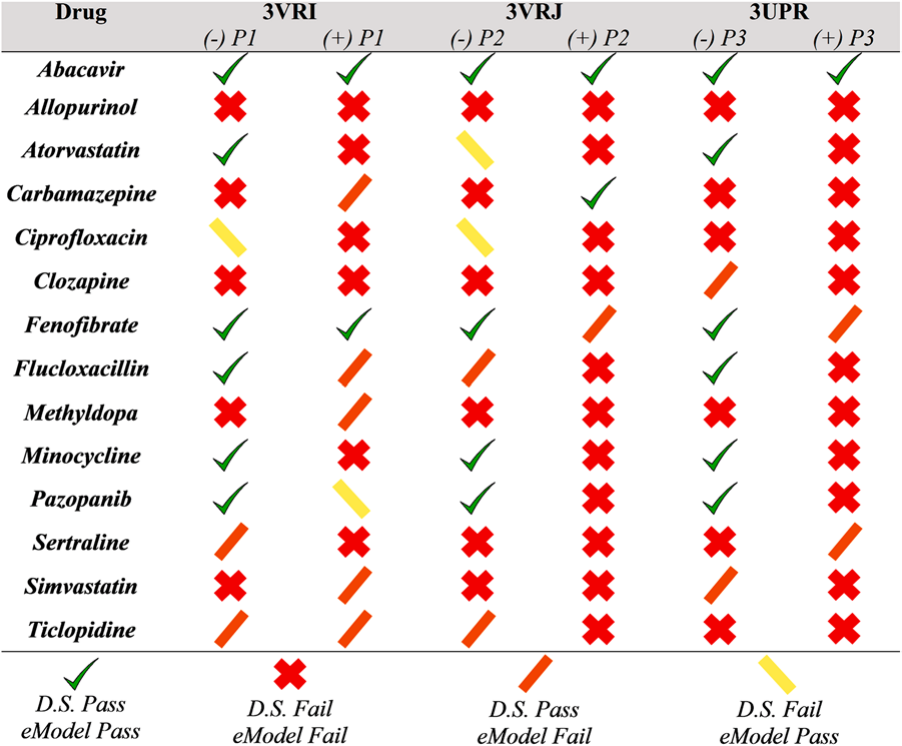
Docking results summary across all three crystals (PDB: 3VRI, 3VRJ, 3UPR) using the XP scoring function with and without peptides P1, P2, and P3

Herein, we conducted a molecular docking study of the HLA-B*57:01 variant using Glide’s SP and XP scoring functions. We were able to self-dock abacavir, an anti-HIV drug known to cause severe ADR via direct binding to B*57:01. After analyzing abacavir’s binding mode to B*57:01 in the presence and the absence of endogenous peptides, we determined that co-binding peptides play a significant role in the binding mode of drugs in HLA antigen binding cleft. Then, we docked a series of drugs known to trigger ADRs via direct HLA interactions and we found that drugs like carbamazepine, fenofibrate, pazopanib, and simvastatin are predicted to have some binding interactions with the HLA-B*57:01 variant. A full summary of our modeling results is provided in Figs. 11 and 12 for both SP and XP scoring functions respectively.

Reliably predicting whether a drug candidate is likely to be an HLA binder at a given variant constitutes a potentially valuable knowledge in evaluating the likelihood of an associated ADR event in a subpopulation of patients. Future work will focus on the B*57:01 screening for the entire DrugBank database [74] which includes over 7000 drugs and drug candidates to identify unknown drug–HLA interactions. Since we showed that endogenous peptides have a significant impact in the binding mode of drugs with HLA, it is still unclear how the peptide changes conformations in response to different drugs, and this is a clear limitation of molecular docking with rigid peptides. Therefore, we plan to conduct peptide self- and cross-docking [68] in the HLA binding cleft as well as molecular dynamics simulations with peptides P1, P2, and P3 to monitor and analyze the dynamic variations of the binding interactions between different peptides, different drugs, and B*57:01. However, as demonstrated in this study, molecular docking represents a fast and reliable cheminformatics technique to forecast drug–HLA interactions. This is critical to screen large datasets of chemicals towards thousands of HLA variants and determine whether a particular drug has a ‘best-binding’ peptide partner that is unique given a particular HLA variant.

## Additional file

**Additional file 1.** Tables showing docking score (TS1) and eModel (TS2) pairwise correlation coefficients between B*57:01 crystals. Figures showing all docking and eModel scores obtained using the 3VRJ and 3UPR crystals (S1–S8).
