# Supplementary material for: Adverse drug reactions triggered by the common HLA-B*57:01 variant: a molecular docking study
Source: J Cheminform. 2017 Mar 4;9:13. doi: 10.1186/s13321-017-0202-6 (PMC5337232; doi:10.1186/s13321-017-0202-6)
Supplement: Supplementary file 1 — Additional file 1. Tables showing docking score (TS1) and eModel (TS2) pairwise correlation coefficients between B*57:01 crystals. Figures showing all docking and eModel scores obtained using the 3VRJ and 3UPR crystals (S1–S8). [file 13321_2017_202_MOESM1_ESM.docx]

**Additional file**

Tables and Figures

| 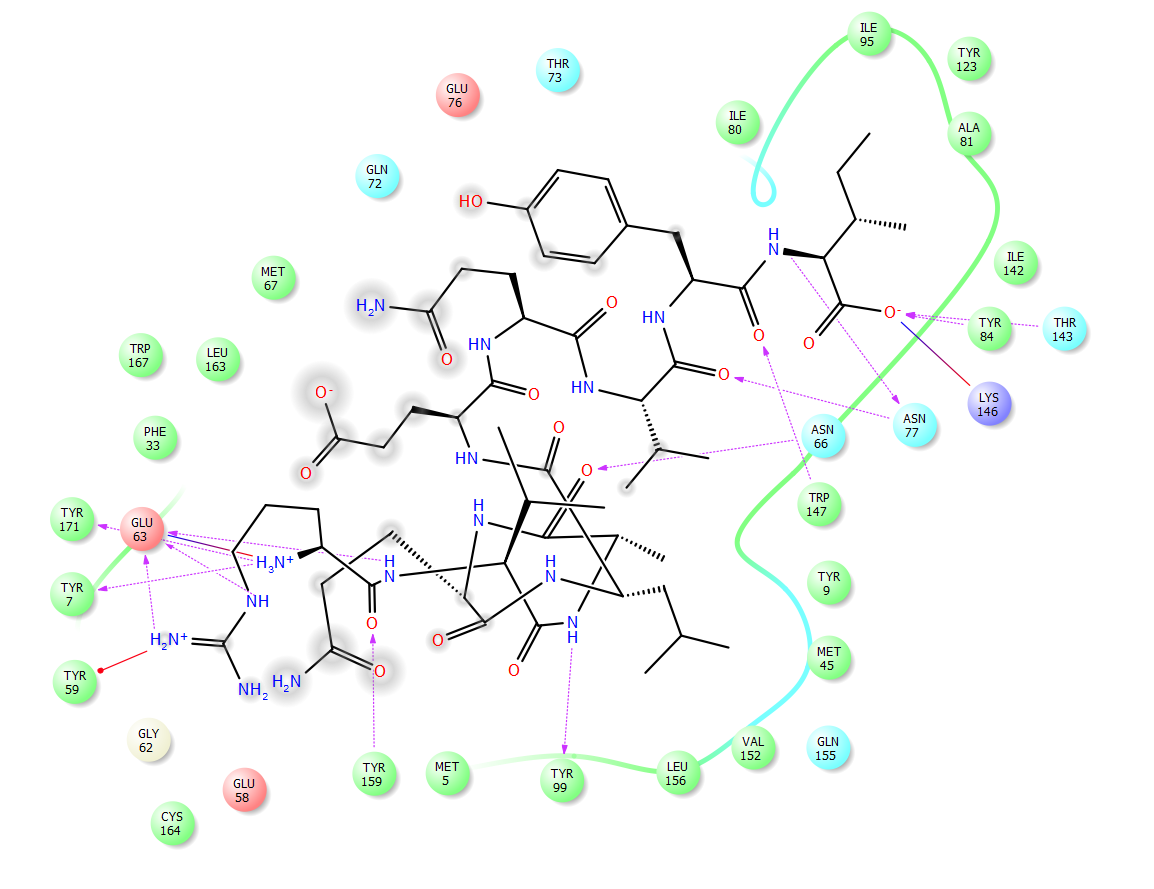 |
| --- |
| A |
| 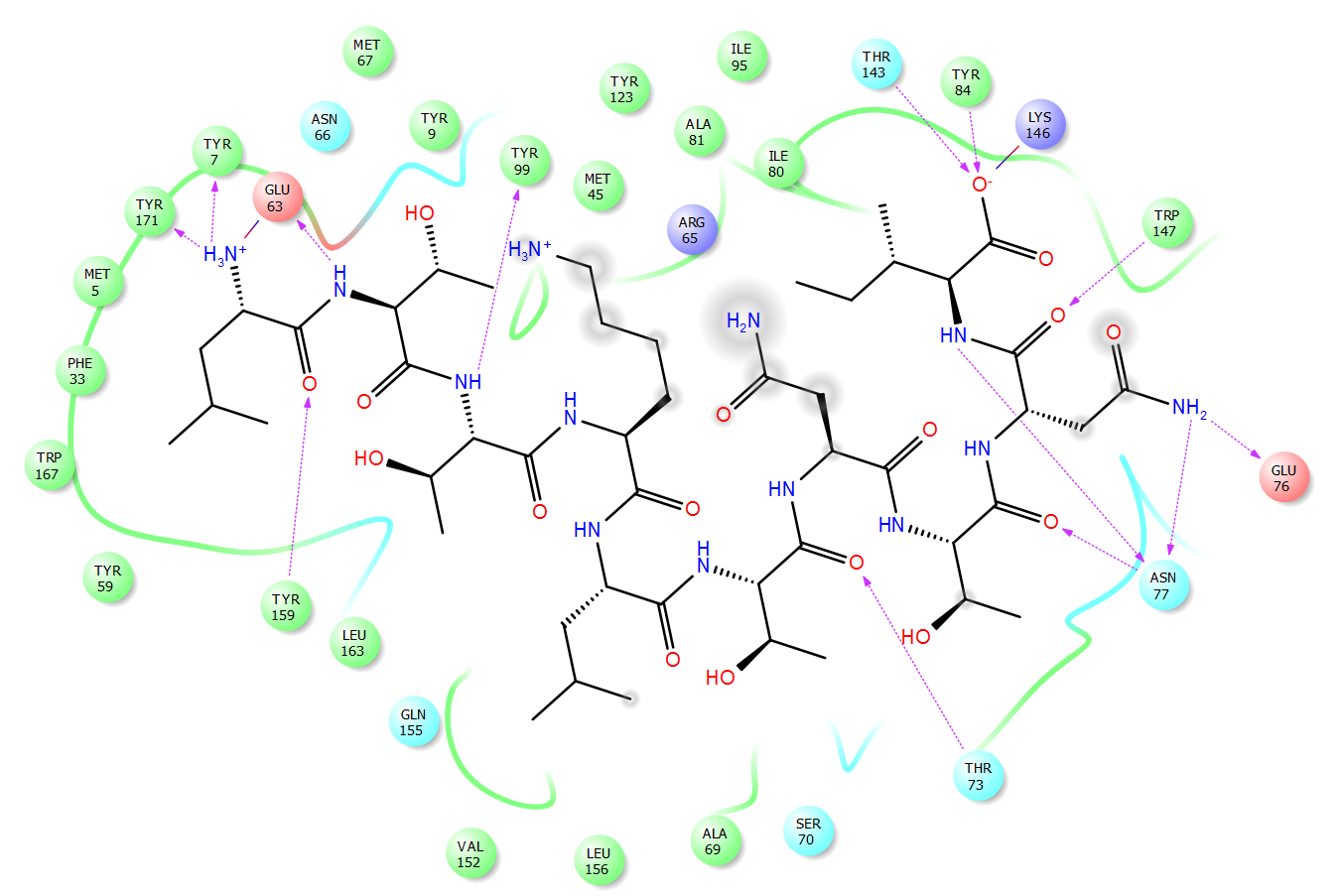 |
| B |
| 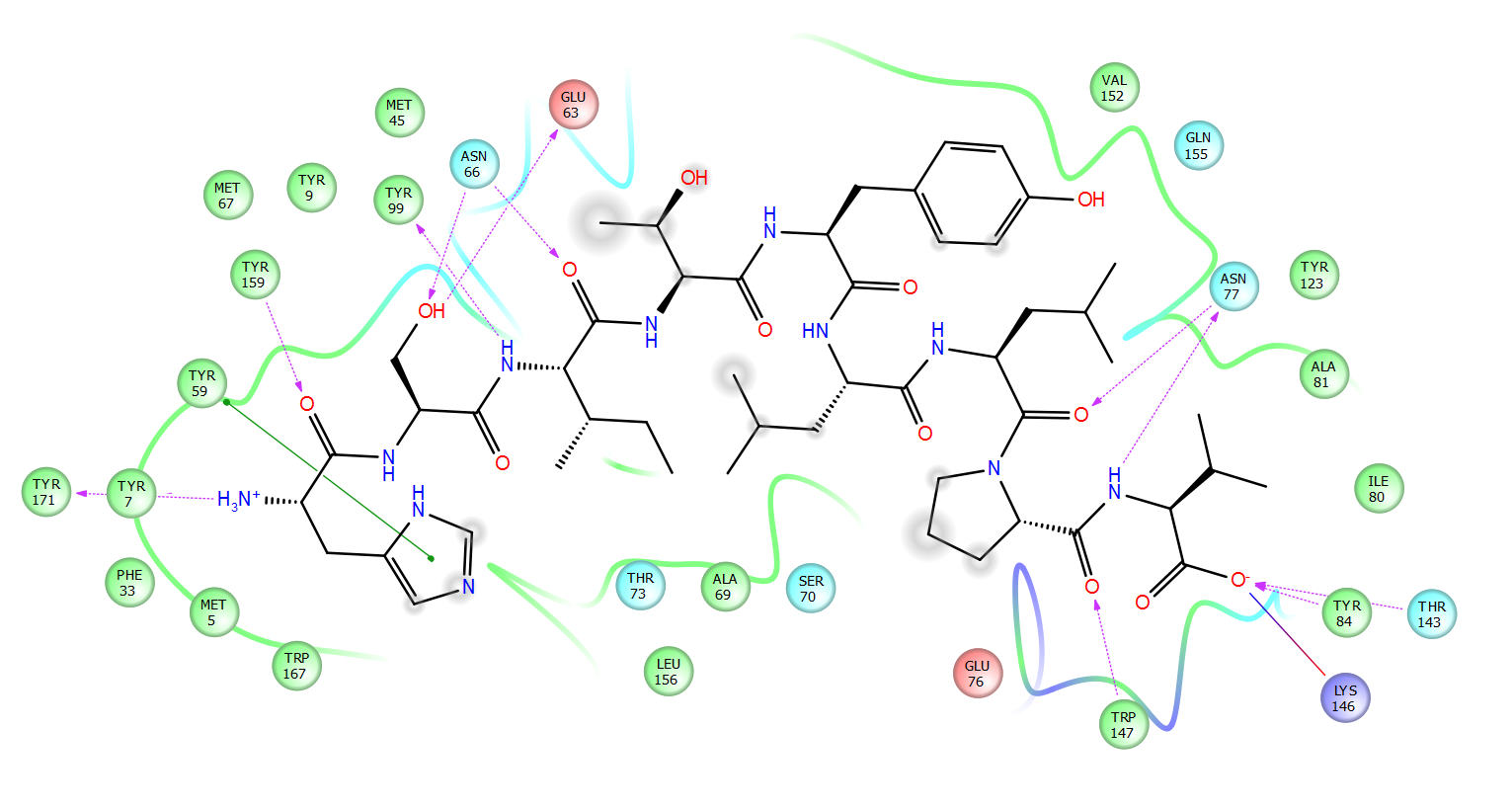 |
| C |

**Figure S1.** Binding mode interaction diagrams of peptides (A) P1, (B) P2, and (C) P3 in the binding pocket of HLA-B*57:01 from X-ray crystals 3VRI, 3VRJ, and 3UPR, respectively.

| 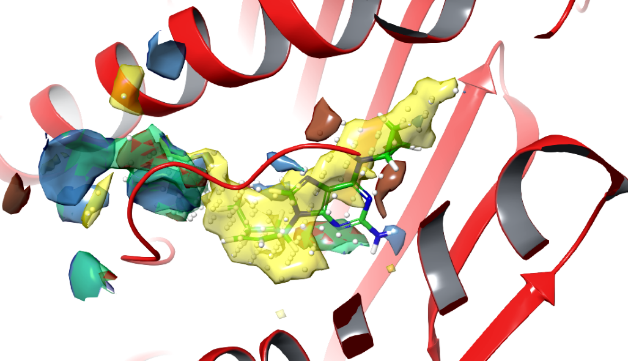 | 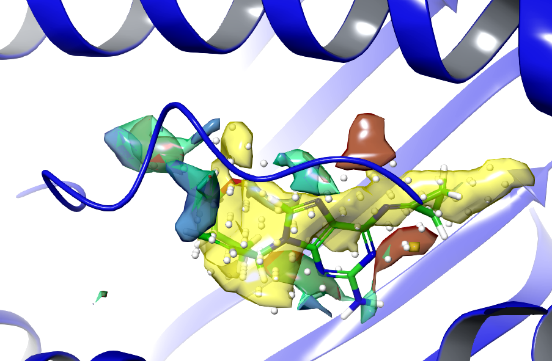 | 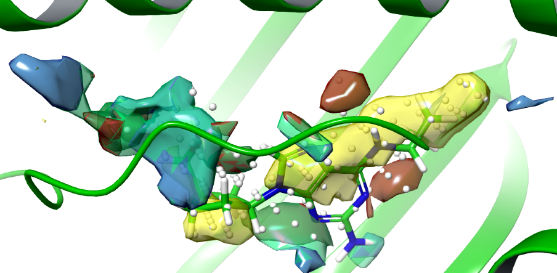 |
| --- | --- | --- |
| A | B | C |
| 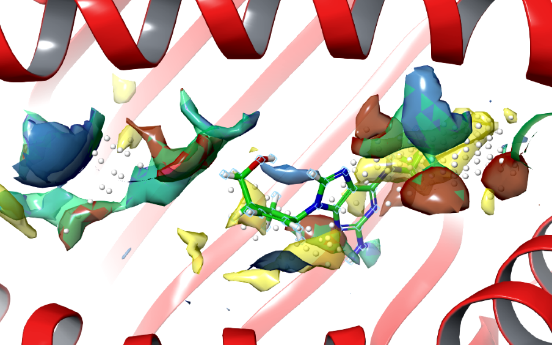 | 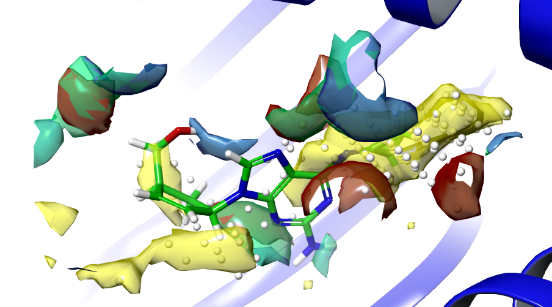 | 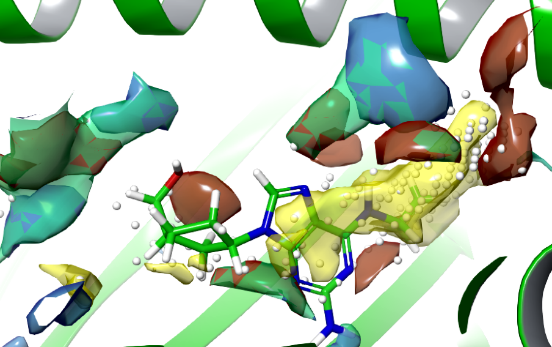 |
| D | E | F |
| 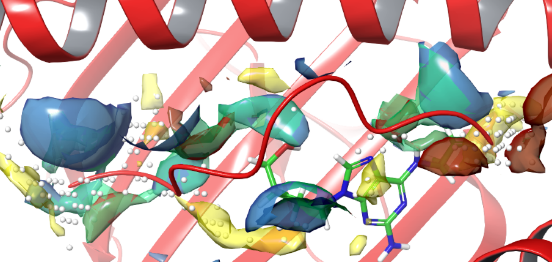 | 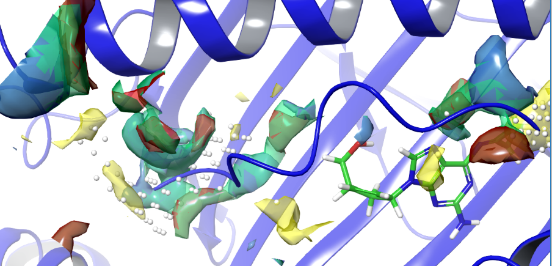 | 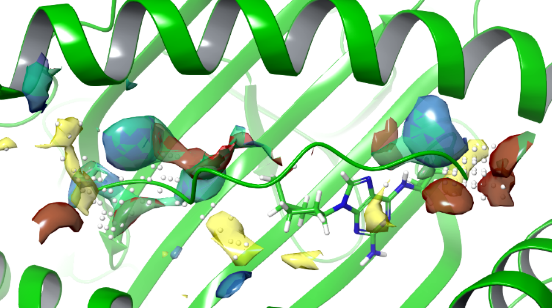 |
| G | H | I |

**Figure S2.** Generated binding site surfaces for 3VRI (red), 3VRJ (dark blue), and 3UPR (dark green) using SiteMap. Hydrophic surfaces are represented in yellow, hydrophilic surfaces are turquoise, H-bond donors are light blue, and H-bond acceptors are brown. SiteMap was run under three conditions: (1) Abacavir as the reference ligand with peptide (A, B, and C), (2) abacavir as the reference ligand without peptide (D, E, and F), and (3) co-binding peptide was used as the reference ligand (G, H, and I).

| 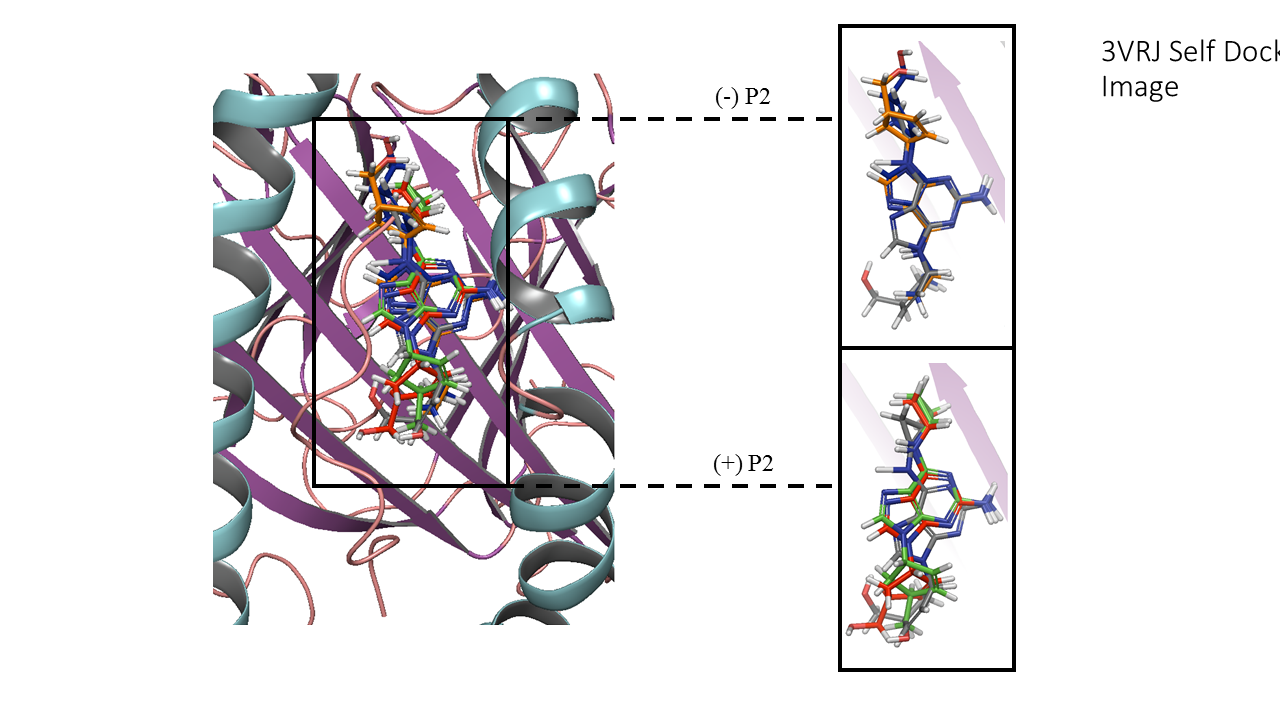 | | |
| --- | --- | --- |
| **Scoring  Function** | **Peptide**  **P2** | **RMSD (Å)** |
| SP | (-) | 7.14 |
|  | (+) | 1.43 |
| XP | (-) | 6.92 |
|  | (+) | 1.83 |

**Figure S3.** Two views of self-docked abacavir with measured RMSD using crystal 3VRJ. 3VRJ native abacavir is shown in gray, abacavir using SP without P2 is shown in blue, abacavir using SP with P2 is shown in red, abacavir using XP without P2 is shown in orange, and abacavir using XP with P2 is shown in green. *Note: SP and XP without P2 docks abacavir with the hydroxyl groups located at opposite ends of the binding site (180 degree rotation)*

| 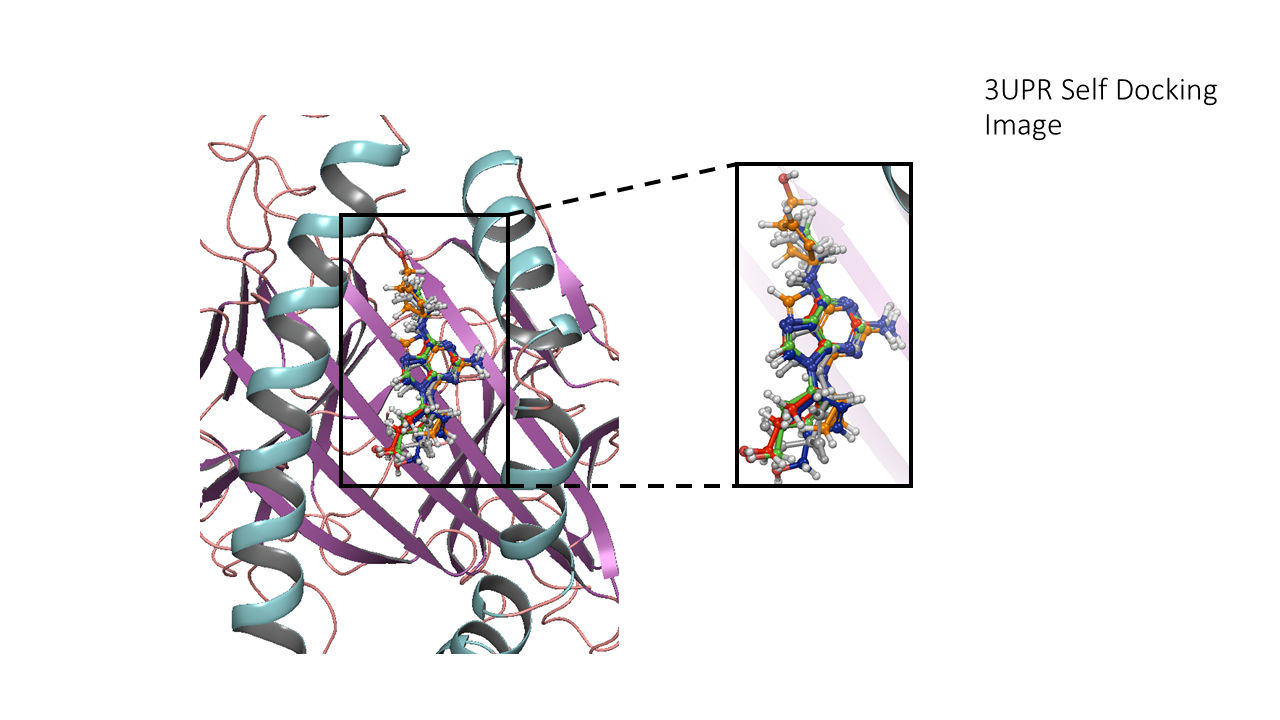 | | |
| --- | --- | --- |
| **Scoring  Function** | **Peptide**  **P3** | **RMSD (Å)** |
| SP | (-) | 0.98 |
|  | (+) | 1.22 |
| XP | (-) | 7.22 |
|  | (+) | 1.19 |

**Figure S4.** Two views of self-docked abacavir with measured RMSD using crystal 3UPR. 3UPR native abacavir is shown in gray, abacavir using SP without P3 is shown in blue, abacavir using SP with P3 is shown in red, abacavir using XP without P3 is shown in orange, and abacavir using XP with P3 is shown in green. *Note: XP without P3 docks abacavir with the hydroxyl groups located at opposite ends of the binding site (180 degree rotation)*

| 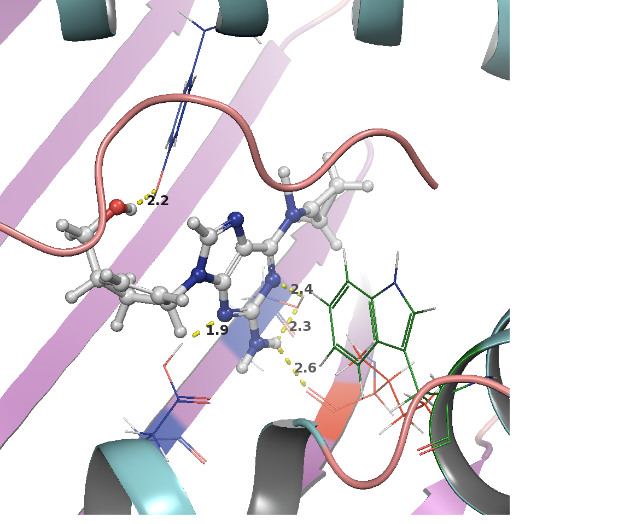 | 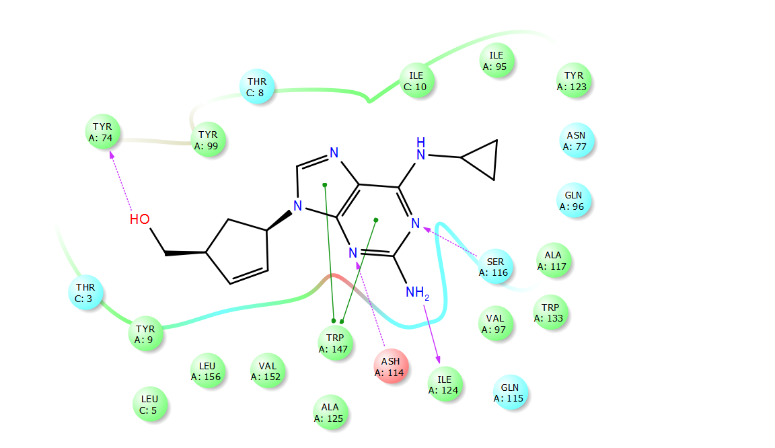 |
| --- | --- |
| A | |
| 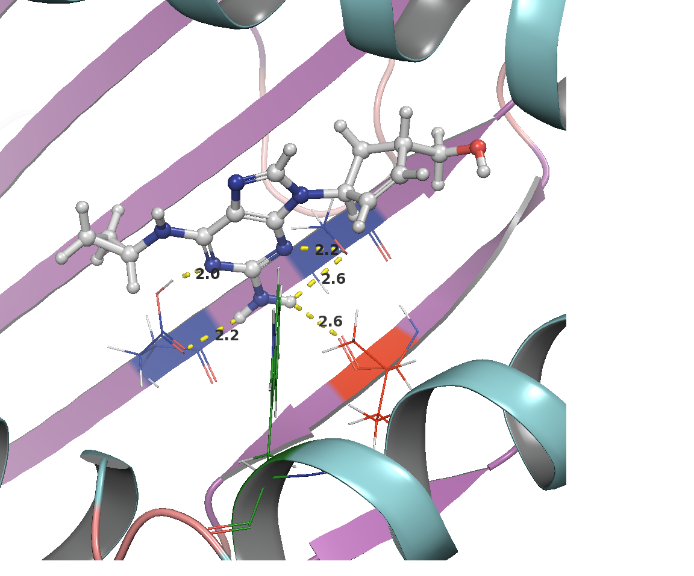 | 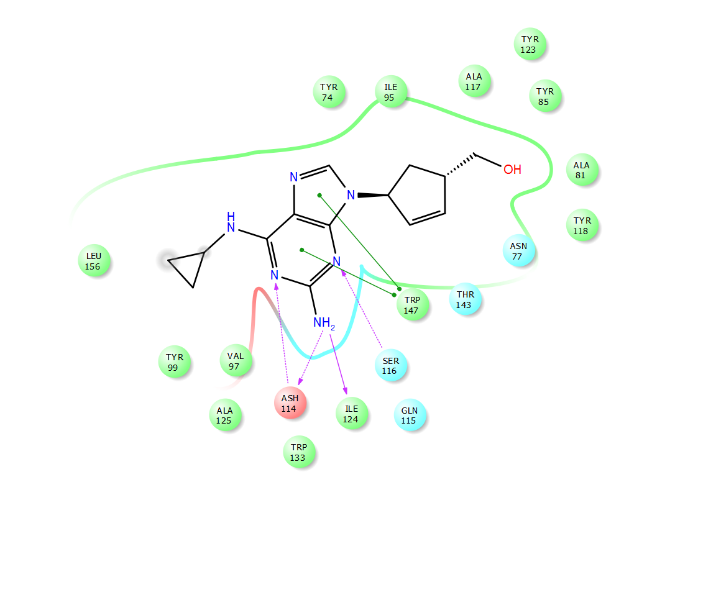 |
| B | |
| 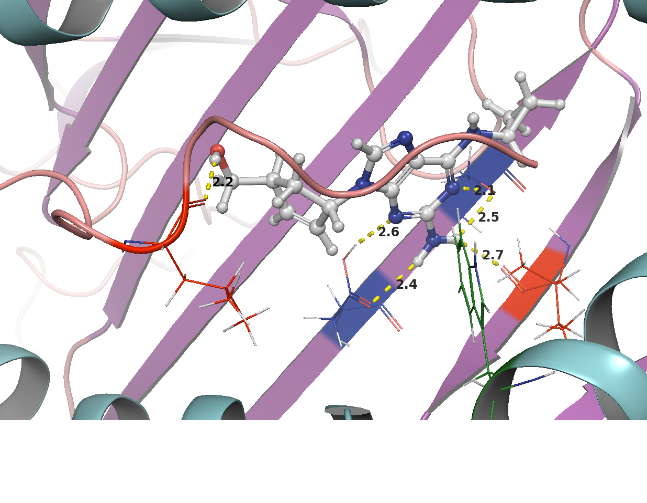 | 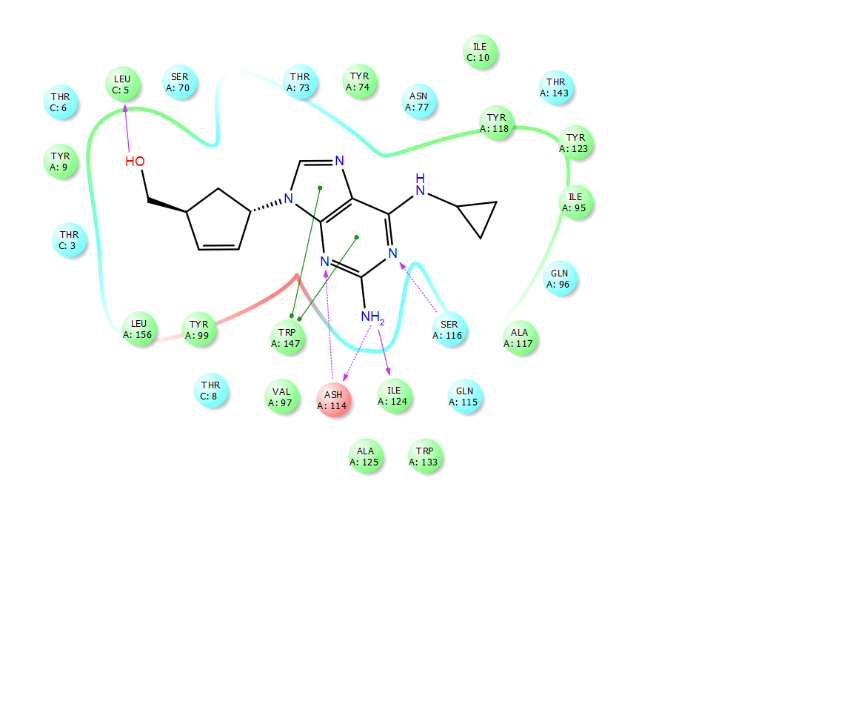 |
| C | |

**Figure S5.** Binding mode interactions of abacavir-B*57:01 found from crystal 3VRJ. **A**. Native abacavir binding mode from X-ray crystal 3VRJ with P2. **B**. Docked binding mode found using SP scoring function withot P2. **C**. Docked binding mode found using SP scoring function with P2. B*57:01 protein is represented as follows: Helix (cyan), sheet (magneta), and loops (salmon). The ligand is colored by element: Carbon (gray), nitrogen (blue), and oxygen (red). Ligand—amino acid interactions are colored as follows: side chain H-bonding (Dark Blue), backbone H-bonding (Red), and π-π stacking (Forest Green).

*Notes: - SP and XP binding modes with P2 are the same.*

- *SP and XP binding modes without P2 are extremely similar. It should be noted that in SP without P2 there are several residues that could possibly contribute to H-bonding with the hydroxyl group but these residues are well beyond 3 Å of the hydroxyl group or are found in a conformation that cannot undergo H-bonding. TYR85 (5.2 Å), ALA81 (6 Å), ASN77 (4.6 Å, incorrect orientation that causes repulsion), TYR118 (6.5 Å).
  The XP results without P2 produced a very similar orientation as the SP without P2. However, there were some slightly different A.A. residues in the proximity of the hydroxyl group that were worth considering, with only one having a potential H-bond potential (TYR123 was with 3 Å of the hydroxyl group). The other A.A. considered were well beyond the 3 Å threshold (TYR 84, TYR 118, ALA81, and ASN 77).*

| 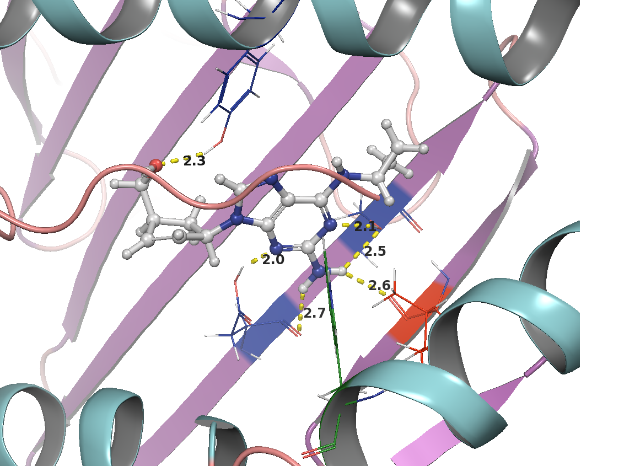 | 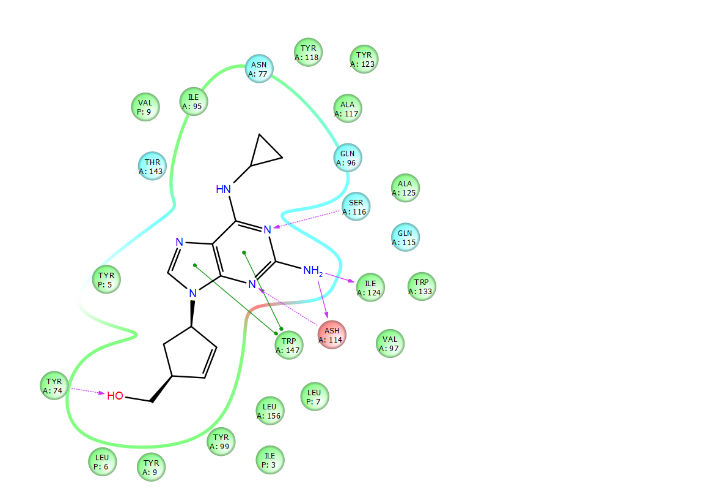 |
| --- | --- |
| A | |
| 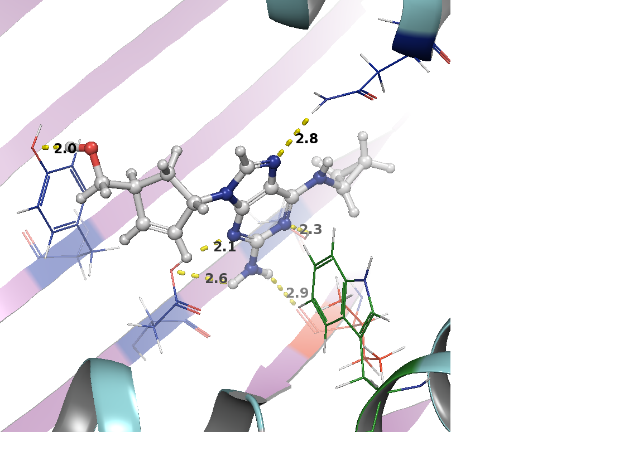 | 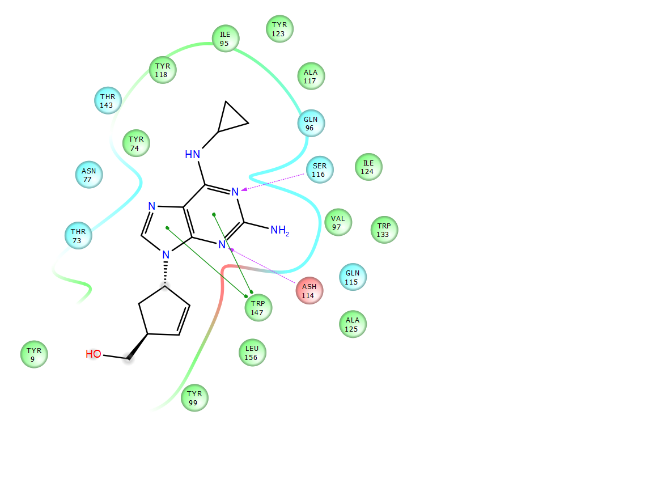 |
| B | |
| 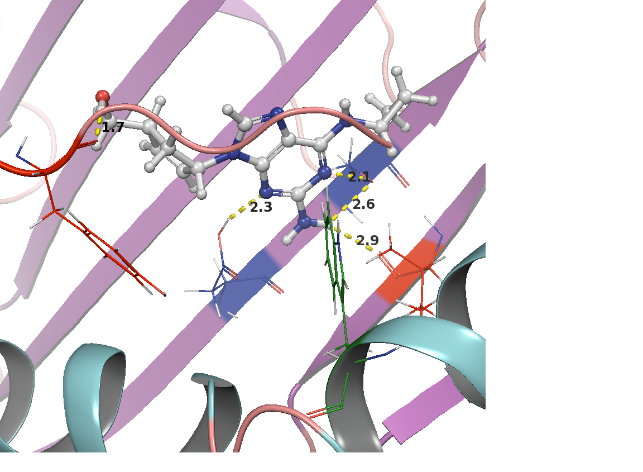 | 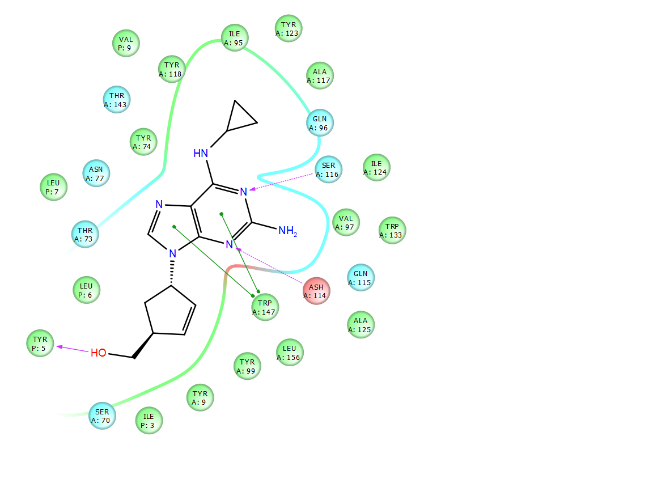 |
| C | |

**Figure S6.** Binding mode interactions of abacavir-B*57:01 found from crystal 3UPR. **A**. Native abacavir binding mode from X-ray crystal 3UPR with P3. **B**. Docked binding mode found using SP scoring function with P3. **C**. Docked binding mode found using SP scoring function without P3. B*57:01 protein is represented as follows: Helix (cyan), sheet (magneta), and loops (salmon). The ligand is colored by element: Carbon (gray), nitrogen (blue), and oxygen (red). Ligand—amino acid interactions are colored as follows: side chain H-bonding (Dark Blue), backbone H-bonding (Red), and π-π stacking (Forest Green). *Notes: XP binding mode without P3 docks abacavir with a rotation of 180 degrees (the hydroxyl group appears where the cyclopropyl moiety should be when compared to the native abacavir. This is similar to the case shown for 3VRJ SP and XP functions without P2. The ILE124 Hbond appears to be on the cusp of the H-bond limit (it is greater than 3 Å from the ligand). Also worth noting, is that depending on where abacavir is placed may or may not allow for H-bonding with the ASN77 residue (as shown in 3D representation of panel B). SP and XP binding modes with P3 produce similar abacavir orientations in the pocket.*

| **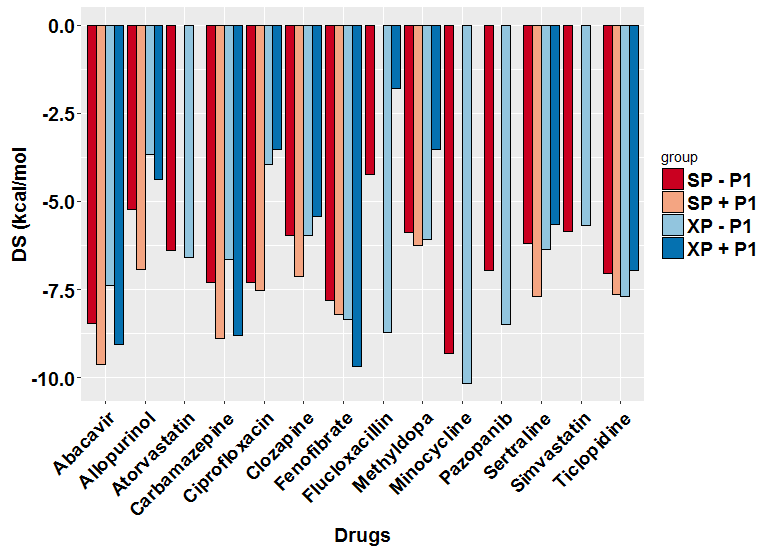** |
| --- |
| **A** |
| **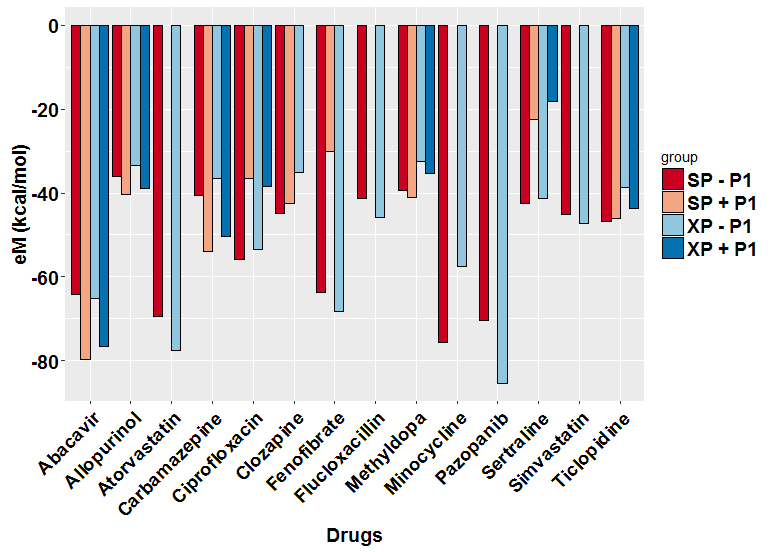** |
| **B** |

**Figure S7.** Docking score and eModel distributions reported as absolute values with threshold scores indicated at 7 kcal/mol (Docking Score) and 50 kcal/mol (eModel) measured using 3VRJ. **A**. Docking score distribution of the test set of compounds. **B**. eModel distribution of the test set of compounds. SP without P2 results are shown in red, SP with P2 results are shown as maroon, XP without P2 results are shown in light blue, and XP with P2 results are shown in dark blue.

| **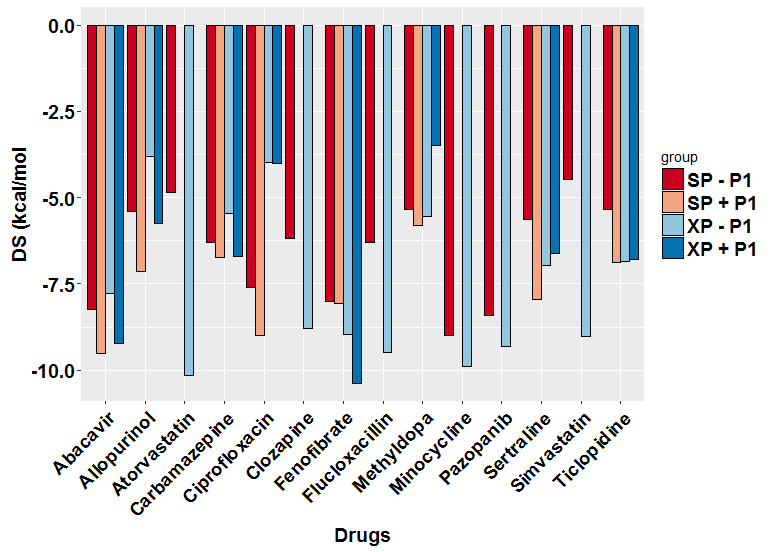** |
| --- |
| **A** |
| **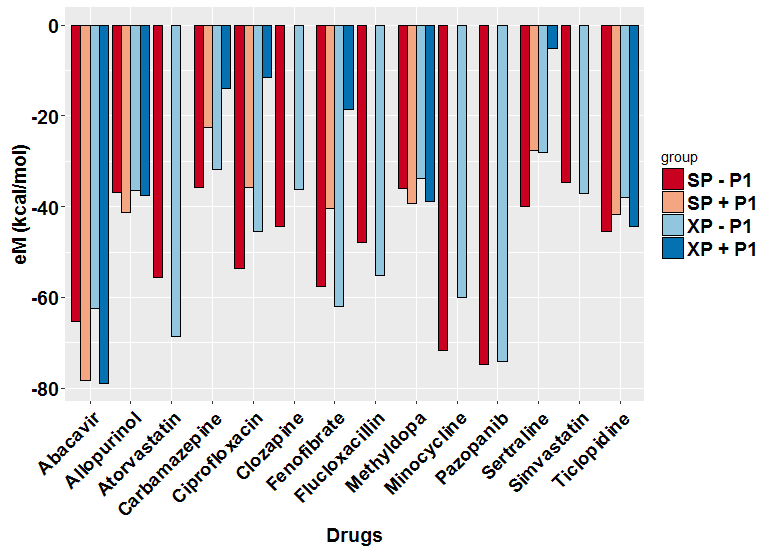** |
| **B** |

**Figure S8.** Docking score and eModel distributions reported as absolute values with threshold scores indicated at 7 kcal/mol (Docking Score) and 50 kcal/mol (eModel) measured using 3UPR. **A**. Docking score distribution of the test set of compounds. **B**. eModel distribution of the test set of compounds. SP without P3 results are shown in red, SP with P3 results are shown as maroon, XP without P3 results are shown in light blue, and XP with P3 results are shown in dark blue.

**
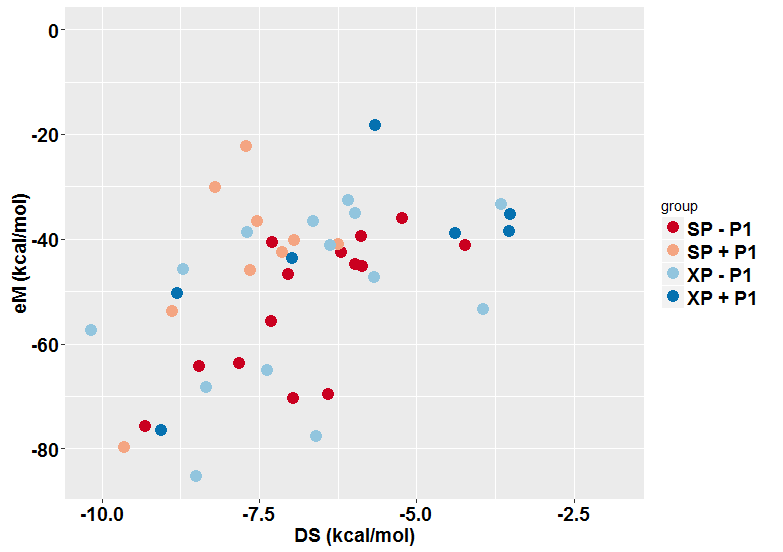
**

**Figure S9.** eModel vs Docking Score plot for full set of test compounds with 3VRJ SP without P2 results are shown in red, SP with P2 results are shown as maroon, XP without P2 results are shown in light blue, and XP with P2 results are shown in dark blue.

**
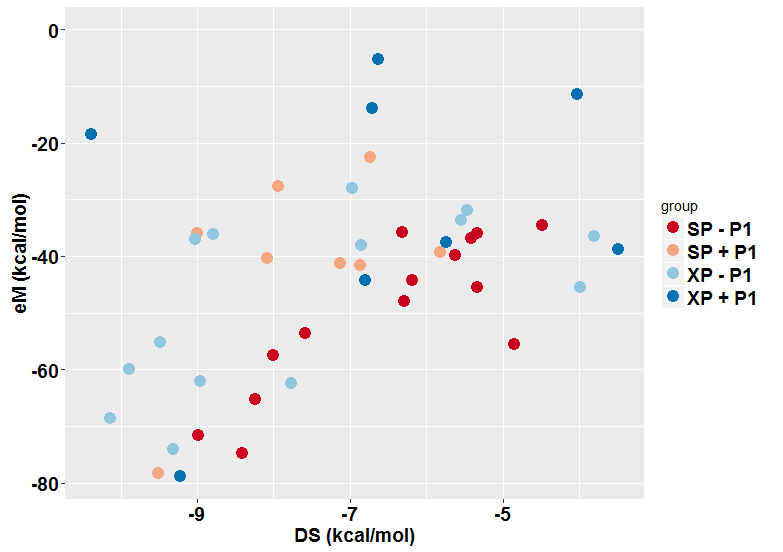
**

**Figure S10.** eModel vs Docking Score plot for full set of test compounds using 3UPR. SP without P3 results are shown in red, SP with P3 results are shown as maroon, XP without P3 results are shown in light blue, and XP with P3 results are shown in dark blue.

**Table S1.** Statistical analysis of molecular docking’s ability to forecast HLA-B*57:01 liable drugs from the test set of compounds.

|  |  | **3VRI** | | | | **3VRJ** | | | | **3UPR** | | | | |
| --- | --- | --- | --- | --- | --- | --- | --- | --- | --- | --- | --- | --- | --- | --- |
|  |  | **(-) P1** | | **(+) P1** | | **(-) P2** | | **(+) P2** | | **(-) P3** | | **(+) P3** | |  |
| **Drugs** | *Expected* | *SP* | *XP* | *SP* | *XP* | *SP* | *XP* | *SP* | *XP* | *SP* | *XP* | *SP* | *XP* |  |
| Abacavir^a^ | 1 | 1 | 1 | 1 | 1 | 1 | 1 | 1 | 1 | 1 | 1 | 1 | 1 |  |
| Allopurinol | 0 | 0 | 0 | 0 | 0 | 0 | 0 | 0 | 0 | 0 | 0 | 0 | 0 |  |
| Atorvastatin | 0 | 0 | 1 | 0 | 0 | 0 | 0 | 0 | 0 | 0 | 1 | 0 | 0 |  |
| Carbamazepine | 0 | 0 | 0 | 0 | 0 | 0 | 0 | 1 | 1 | 0 | 0 | 0 | 0 |  |
| Ciprofloxacin | 0 | 1 | 0 | 0 | 0 | 1 | 0 | 0 | 0 | 1 | 0 | 0 | 0 |  |
| Clozapine | 0 | 0 | 0 | 0 | 0 | 0 | 0 | 0 | 0 | 0 | 0 | 0 | 0 |  |
| Fenofibrate | 0 | 1 | 1 | 1 | 1 | 1 | 1 | 0 | 0 | 1 | 1 | 0 | 0 |  |
| Flucloxacillin^b^ | 1 | 0 | 1 | 0 | 0 | 0 | 0 | 0 | 0 | 0 | 1 | 0 | 0 |  |
| Methyldopa | 0 | 0 | 0 | 0 | 0 | 0 | 0 | 0 | 0 | 0 | 0 | 0 | 0 |  |
| Minocycline | 0 | 1 | 1 | 0 | 0 | 1 | 1 | 0 | 0 | 1 | 1 | 0 | 0 |  |
| Pazopanib^c^ | 1 | 1 | 1 | 0 | 0 | 1 | 1 | 0 | 0 | 1 | 1 | 0 | 0 |  |
| Sertraline | 0 | 0 | 0 | 0 | 0 | 0 | 0 | 0 | 0 | 0 | 0 | 0 | 0 |  |
| Simvastatin | 0 | 0 | 0 | 0 | 0 | 0 | 0 | 0 | 0 | 0 | 0 | 0 | 0 |  |
| Ticlopidine | 0 | 0 | 0 | 0 | 0 | 0 | 0 | 0 | 0 | 0 | 0 | 0 | 0 |  |
| **TP** | 3 | 2 | 3 | 1 | 1 | 1 | 2 | 1 | 1 | 2 | 3 | 1 | 1 |  |
| **FP** | 0 | 3 | 3 | 1 | 1 | 3 | 2 | 1 | 1 | 3 | 3 | 0 | 0 |  |
| **TN** | 11 | 8 | 8 | 10 | 10 | 8 | 9 | 10 | 10 | 8 | 8 | 11 | 11 |  |
| **FN** | 0 | 1 | 0 | 2 | 2 | 2 | 1 | 2 | 2 | 1 | 0 | 2 | 2 |  |
| **Accuracy** |  | 0.71 | 0.79 | 0.79 | 0.79 | 0.71 | 0.79 | 0.79 | 0.79 | 0.71 | 0.79 | 0.86 | 0.86 |  |
| **Sensitivity** |  | 0.67 | 1.00 | 0.33 | 0.33 | 0.67 | 0.67 | 0.33 | 0.33 | 0.67 | 1.00 | 0.33 | 0.33 |  |
| **Specificity** |  | 0.73 | 0.73 | 0.91 | 0.91 | 0.73 | 0.82 | 0.91 | 0.91 | 0.73 | 0.73 | 1.00 | 1.00 |  |
| **PPV** |  | 0.40 | 0.50 | 0.50 | 0.50 | 0.40 | 0.50 | 0.50 | 0.50 | 0.40 | 0.50 | 1.00 | 1.00 |  |
| **NPV** |  | 0.89 | 1.00 | 0.83 | 0.83 | 0.89 | 0.90 | 0.83 | 0.83 | 0.89 | 1.00 | 0.85 | 0.85 |  |
|  | 1. See ref. [1–5] | | | | | | | | | | | | |  |
|  | 1. See ref. [6] 2. See ref. [7] | | | | | | | | | | | | |  |

**Table S1 Notes:**

Values have been binned where a 1 represents the drug is HLA-B*57:01 liable (in the case of our model it passed both the DS and eM scoring thresholds) a 0 represents the drug is not HLA-B*57:01 liable (it failed one or more of the criteria for activity within our model).

True positives (TP), false positives (FP), true negatives (TN), and false negatives (FN) were determined based on their expected activity. These were then used to calculate the accuracy, sensitivity, specificity, positive prediction value (PPV), and negative prediction value (NPV) [8]. We measured these statistical parameters for thoroughness, but it is important to remember that this model was developed for qualitative predictions of HLA-B*57:01 activity and not quantitative predictions.

Accuracy was measured using the following equation:

$TP= \frac{TP+TN}{TP+FP+TN+FN}$.

Sensitivity was measured using the following equation,

$Sensitivity= \frac{\mathrm{TP}}{TP+FN}$.

Specificity was measured using the following equation,

$Specificity= \frac{\mathrm{TN}}{FP+TN}$.

PPV was calculated using the following equation,

$PPV= \frac{\mathrm{TP}}{TP+FP}$.

NPV was calculated using the following equation,

$NPV= \frac{TN}{TN+FN}$.

**Table S2.** Pairwise tanimoto similarities of the test set of compounds measured using MACCS fingerprints.

| **Drugs** | **Aba** | **Allo** | **Ato** | **Car** | **Cip** | **Clo** | **Fen** | **Flu** | **Met** | **Min** | **Paz** | **Ser** | **Sim** | **Tic** |
| --- | --- | --- | --- | --- | --- | --- | --- | --- | --- | --- | --- | --- | --- | --- |
| **Abacavir**  **(Aba)** | 1.00 | 0.48 | 0.48 | 0.43 | 0.55 | 0.49 | 0.15 | 0.35 | 0.33 | 0.34 | 0.43 | 0.38 | 0.23 | 0.42 |
| **Allopurinol**  **(All)** | 0.48 | 1.00 | 0.35 | 0.39 | 0.41 | 0.32 | 0.19 | 0.44 | 0.29 | 0.34 | 0.44 | 0.21 | 0.17 | 0.30 |
| **Atorvastatin**  **(Ato)** | 0.48 | 0.35 | 1.00 | 0.29 | 0.63 | 0.46 | 0.33 | 0.52 | 0.39 | 0.50 | 0.31 | 0.38 | 0.37 | 0.41 |
| **Carbamazepine**  **(Car)** | 0.43 | 0.39 | 0.29 | 1.00 | 0.32 | 0.38 | 0.17 | 0.28 | 0.25 | 0.33 | 0.38 | 0.30 | 0.17 | 0.33 |
| **Ciprofloxacin**  **(Cip)** | 0.55 | 0.41 | 0.63 | 0.32 | 1.00 | 0.56 | 0.32 | 0.49 | 0.40 | 0.43 | 0.33 | 0.37 | 0.37 | 0.49 |
| **Clozapine**  **(Clo)** | 0.49 | 0.32 | 0.46 | 0.38 | 0.56 | 1.00 | 0.19 | 0.32 | 0.22 | 0.29 | 0.36 | 0.53 | 0.22 | 0.63 |
| **Fenofibrate**  **(Fen)** | 0.15 | 0.19 | 0.33 | 0.17 | 0.32 | 0.19 | 1.00 | 0.39 | 0.44 | 0.38 | 0.18 | 0.28 | 0.41 | 0.20 |
| **Flucloxacillin**  **(Flu)** | 0.35 | 0.44 | 0.52 | 0.28 | 0.49 | 0.32 | 0.39 | 1.00 | 0.36 | 0.45 | 0.40 | 0.28 | 0.30 | 0.32 |
| **Methyldopa**  **(Met)** | 0.33 | 0.29 | 0.39 | 0.25 | 0.40 | 0.22 | 0.44 | 0.36 | 1.00 | 0.51 | 0.20 | 0.34 | 0.36 | 0.20 |
| **Minocycline**  **(Min)** | 0.34 | 0.34 | 0.50 | 0.33 | 0.43 | 0.29 | 0.38 | 0.45 | 0.51 | 1.00 | 0.30 | 0.31 | 0.44 | 0.20 |
| **Pazopanib**  **(Paz)** | 0.43 | 0.44 | 0.31 | 0.38 | 0.33 | 0.36 | 0.18 | 0.40 | 0.20 | 0.30 | 1.00 | 0.25 | 0.16 | 0.32 |
|  |  |  |  |  |  |  |  |  |  |  |  |  |  |  |
|  |  |  |  |  |  |  |  |  |  |  |  |  |  |  |
| **Drugs** | **Aba** | **All** | **Ato** | **Car** | **Cip** | **Clo** | **Fen** | **Flu** | **Met** | **Min** | **Paz** | **Ser** | **Sim** | **Tic** |
| **Sertraline**  **(Set)** | 0.38 | 0.21 | 0.38 | 0.30 | 0.37 | 0.53 | 0.28 | 0.28 | 0.34 | 0.31 | 0.25 | 1.00 | 0.24 | 0.46 |
| **Simvastatin**  **(Sim)** | 0.23 | 0.17 | 0.37 | 0.17 | 0.37 | 0.22 | 0.41 | 0.30 | 0.36 | 0.44 | 0.16 | 0.24 | 1.00 | 0.20 |
| **Ticlopidine**  **(Tic)** | 0.42 | 0.30 | 0.41 | 0.33 | 0.49 | 0.63 | 0.20 | 0.32 | 0.20 | 0.20 | 0.32 | 0.46 | 0.20 | 1.00 |

**Table S3.** Docking score Pearson correlation coefficients between B*57:01 crystals and model parameters.

|  |  |  | **3VRI** | | | | **3VRJ** | | | | **3UPR** | | | |
| --- | --- | --- | --- | --- | --- | --- | --- | --- | --- | --- | --- | --- | --- | --- |
|  |  |  | **(-) P1** | | **(+) P1** | | **(-) P2** | | **(+) P2** | | **(-) P3** | | **(+) P3** | |
|  |  |  | *SP* | *XP* | *SP* | *XP* | *SP* | *XP* | *SP* | *XP* | *SP* | *XP* | *SP* | *XP* |
| **3VRI** | **(-) P1** | *SP* | 1.00 |  |  |  |  |  |  |  |  |  |  |  |
|  |  | *XP* | 0.75 | 1.00 |  |  |  |  |  |  |  |  |  |  |
|  | **(+) P1** | *SP* | 0.87 | 0.75 | 1.00 |  |  |  |  |  |  |  |  |  |
|  |  | *XP* | 0.51 | 0.43 | 0.74 | 1.00 |  |  |  |  |  |  |  |  |
| **3VRJ** | **(-) P2** | *SP* | **0.88** | 0.64 | 0.86 | 0.64 | 1.00 |  |  |  |  |  |  |  |
|  |  | *XP* | 0.74 | **0.98** | 0.78 | 0.50 | 0.71 | 1.00 |  |  |  |  |  |  |
|  | **(+) P2** | *SP* | 0.80 | 0.58 | **0.87** | 0.63 | 0.84 | 0.61 | 1.00 |  |  |  |  |  |
|  |  | *XP* | 0.40 | 0.26 | 0.73 | **0.70** | 0.51 | 0.31 | 0.63 | 1.00 |  |  |  |  |
| **3UPR** | **(-) P3** | *SP* | **0.82** | 0.64 | 0.74 | 0.50 | **0.71** | 0.68 | 0.67 | 0.36 | 1.00 |  |  |  |
|  |  | *XP* | 0.77 | **0.92** | 0.69 | 0.41 | 0.63 | **0.92** | 0.60 | 0.42 | 0.83 | 1.00 |  |  |
|  | **(+) P3** | *SP* | 0.65 | 0.75 | **0.57** | 0.27 | 0.64 | 0.79 | **0.61** | 0.27 | 0.81 | 0.85 | 1.00 |  |
|  |  | *XP* | 0.41 | 0.55 | 0.68 | **0.74** | 0.47 | 0.60 | 0.43 | **0.74** | 0.50 | 0.73 | 0.54 | 1.00 |

**Table S4.** eModel score Pearson correlation coefficients between B*57:01 crystals and model parameters.

|  |  |  | **3VRI** | | | | **3VRJ** | | | | **3UPR** | | | |
| --- | --- | --- | --- | --- | --- | --- | --- | --- | --- | --- | --- | --- | --- | --- |
|  |  |  | ***(-) P1*** | | ***(+) P1*** | | ***(-) P2*** | | ***(+) P2*** | | ***(-) P3*** | | ***(+) P3*** | |
|  |  |  | *SP* | *XP* | *SP* | *XP* | *SP* | *XP* | *SP* | *XP* | *SP* | *XP* | *SP* | *XP* |
| **3VRI** | ***(-) P1*** | *SP* | 1.00 |  |  |  |  |  |  |  |  |  |  |  |
|  |  | *XP* | 0.64 | 1.00 |  |  |  |  |  |  |  |  |  |  |
|  | ***(+) P1*** | *SP* | 0.33 | 0.19 | 1.00 |  |  |  |  |  |  |  |  |  |
|  |  | *XP* | 0.07 | 0.23 | -0.04 | 1.00 |  |  |  |  |  |  |  |  |
| **3VRJ** | ***(-) P2*** | *SP* | **0.84** | 0.51 | 0.60 | 0.11 | 1.00 |  |  |  |  |  |  |  |
|  |  | *XP* | 0.65 | **0.97** | 0.21 | 0.26 | 0.43 | 1.00 |  |  |  |  |  |  |
|  | ***(+) P2*** | *SP* | 0.39 | 0.32 | **0.60** | 0.73 | 0.34 | 0.48 | 1.00 |  |  |  |  |  |
|  |  | *XP* | -0.33 | -0.12 | 0.09 | **0.83** | -0.35 | 0.06 | 0.41 | 1.00 |  |  |  |  |
| **3UPR** | ***(-) P3*** | *SP* | **0.74** | 0.58 | 0.52 | -0.37 | **0.73** | 0.57 | 0.20 | -0.65 | 1.00 |  |  |  |
|  |  | *XP* | 0.68 | **0.94** | 0.21 | 0.22 | 0.56 | **0.93** | 0.26 | -0.14 | 0.62 | 1.00 |  |  |
|  | ***(+) P3*** | *SP* | 0.82 | 0.62 | **0.65** | 0.71 | 0.72 | 0.71 | **0.91** | -0.17 | 0.83 | 0.68 | 1.00 |  |
|  |  | *XP* | -0.01 | 0.16 | 0.43 | **0.70** | 0.23 | 0.21 | 0.22 | **0.67** | 0.21 | 0.46 | 0.25 | 1.00 |

References:

1. Illing PT, Vivian JP, Dudek NL, Kostenko L, Chen Z, Bharadwaj M, et al. Immune self-reactivity triggered by drug-modified HLA-peptide repertoire. Nature. Nature Publishing Group; 2012;486:554–8.

2. Ostrov D a., Grant BJ, Pompeu Y a., Sidney J, Harndahl M, Southwood S, et al. Drug hypersensitivity caused by alteration of the MHC-presented self-peptide repertoire. Proc. Natl. Acad. Sci. 2012;109:9959–64.

3. Martin AM, Nolan D, Gaudieri S, Almeida CA, Nolan R, James I, et al. Predisposition to abacavir hypersensitivity conferred by HLA-B*5701 and a haplotypic Hsp70-Hom variant. Proc. Natl. Acad. Sci. U. S. A. 2004;101:4180–5.

4. Saag M, Balu R, Phillips E, Brachman P, Martorell C, Burman W, et al. High sensitivity of human leukocyte antigen-b*5701 as a marker for immunologically confirmed abacavir hypersensitivity in white and black patients. Clin. Infect. Dis. 2008;46:1111–8.

5. Berka N, Gill JM, Liacini A, O’Bryan T, Khan FM. Human leukocyte antigen (HLA) and pharmacogenetics: Screening for HLA-B*57:01 among human immunodeficiency virus-positive patients from southern Alberta. Hum. Immunol. Elsevier Inc.; 2012;73:164–7.

6. Daly AK, Donaldson PT, Bhatnagar P, Shen Y, Pe’er I, Floratos A, et al. HLA-B*5701 genotype is a major determinant of drug-induced liver injury due to flucloxacillin. Nat. Genet. Nature Publishing Group; 2009;41:816–9.

7. Xu C-F, Johnson T, Wang X, Carpenter C, Graves AP, Warren L, et al. HLA-B∗57:01 confers susceptibility to pazopanib-associated liver injury in patients with cancer. Clin. Cancer Res. 2016;22:1371–7.

8. Fawcett T. An introduction to ROC analysis Tom. Pattern Recognit. 2006;27:861–74.
